# Realizing quantum convolutional neural networks on a superconducting quantum processor to recognize quantum phases

**DOI:** 10.1038/s41467-022-31679-5

**Published:** 2022-07-16

**Authors:** Johannes Herrmann, Sergi Masot Llima, Ants Remm, Petr Zapletal, Nathan A. McMahon, Colin Scarato, François Swiadek, Christian Kraglund Andersen, Christoph Hellings, Sebastian Krinner, Nathan Lacroix, Stefania Lazar, Michael Kerschbaum, Dante Colao Zanuz, Graham J. Norris, Michael J. Hartmann, Andreas Wallraff, Christopher Eichler

**Affiliations:** 1 Department of Physics, ETH Zurich, CH-8093 Zurich, Switzerland; 2 Department of Physics, Friedrich-Alexander University Erlangen-Nürnberg (FAU), Erlangen, Germany; 3 Quantum Center, ETH Zurich, CH-8093 Zurich, Switzerland

**Keywords:** Quantum information, Qubits, Phase transitions and critical phenomena

## Abstract

Quantum computing crucially relies on the ability to efficiently characterize the quantum states output by quantum hardware. Conventional methods which probe these states through direct measurements and classically computed correlations become computationally expensive when increasing the system size. Quantum neural networks tailored to recognize specific features of quantum states by combining unitary operations, measurements and feedforward promise to require fewer measurements and to tolerate errors. Here, we realize a quantum convolutional neural network (QCNN) on a 7-qubit superconducting quantum processor to identify symmetry-protected topological (SPT) phases of a spin model characterized by a non-zero string order parameter. We benchmark the performance of the QCNN based on approximate ground states of a family of cluster-Ising Hamiltonians which we prepare using a hardware-efficient, low-depth state preparation circuit. We find that, despite being composed of finite-fidelity gates itself, the QCNN recognizes the topological phase with higher fidelity than direct measurements of the string order parameter for the prepared states.

## Abstract (web summary)

Quantum neural networks could help analysing the output of quantum computers and quantum simulators of growing complexity. Here, the authors use a 7-qubit superconducting quantum processor to show how a quantum convolutional neural network can correctly recognise the phase of a quantum many-body state.

## Introduction

Remarkable progress in building quantum hardware^1–4^ has fueled the search for potential applications of both near-term and future error-corrected quantum computers^5,6^, particularly in the simulation of quantum many-body systems^7,8^ and in machine learning^9–12^. For example, the ability of quantum computers to perform linear algebraic operations more efficiently could provide potential speedups for classical machine learning tasks, such as the ordinary matrix inversion in linear regression models^13^. However, dedicated quantum algorithms for this purpose, such as the Harrow, Hassidim and Lloyd algorithm^14^, rely both on executing deep quantum circuits^15^ and on loading binary data into a quantum register^16^ to offer practical advantages, which is beyond the reach of currently available quantum hardware. To load classical data into a quantum register in a more resource-efficient manner and map their features into the high-dimensional Hilbert space to ease classification, quantum circuits parameterized by the input data have been devised and used in quantum support vector machines^17,18^ and quantum neural networks^19^. However, independent of the specific data embedding scheme, it is still an open question whether tasks aiming at the analysis of classical data can ever fully leverage a quantum computer’s capability to process classically unrepresentable amounts of data^9^.

Promising candidates to harness the capabilities of near-term quantum computers are therefore algorithms which process quantum data directly and for which there is no classical analog^9,20^. Quantum computers are beginning to reach a level at which their output states are too complex to be analyzed by classical means^1^, suggesting that machine learning techniques which directly process quantum data are expected to become an increasingly important tool to efficiently characterize and benchmark quantum hardware. Examples of specific applications thereof include the principal component analysis of density matrices^21^, quantum autoencoders^22–24^, the certification of Hamiltonian dynamics^25,26^, and the detection of entanglement correlations in quantum many-body states^10,11,27–29^.

In this work, we experimentally demonstrate the classification of quantum states with quantum neural networks^11^ by implementing a quantum algorithm designed to recognize signatures of topological quantum phases^30–32^. This challenging task is of great relevance for the study of quantum many-body systems^33^ such as high-temperature superconductors^34^. Previous work in this context has focused on recognizing topological quantum phases from (simulated) measurement data using classical machine learning techniques^35–38^. Furthermore, topological states have recently been prepared on quantum hardware and analyzed by measuring characteristic observables^39–41^ such as string order parameters. Here, we experimentally demonstrate a new paradigm to detect symmetry-protected topological states on a 7-qubit quantum device by preparing quantum states within and outside of the SPT phase and by further processing these states with a quantum convolutional neural network to perform quantum phase recognition. We have designed and experimentally implemented a QCNN that, despite being composed of finite-fidelity gates itself, outperforms the direct measurement of the string order parameter in correctly identifying the topological phase. This enhanced capability is achieved by constructing the QCNN to simultaneously measure a weighted sum of Pauli strings, the number of which grows exponentially with the system size *N* (see Supplementary Note 5). The structure of this effectively measured observable allows the QCNN to tolerate both *X*- and *Z*-type errors while processing weakly perturbed input states. Using the QCNN to measure this observable circumvents the need to measure its constituent terms individually.

## Results

### Model

As a model system we consider a family of cluster-Ising Hamiltonians^42^:

1 $$H=-\mathop{\sum }\limits_{i=1}^{N}\left({Z}_{i-1}{X}_{i}{Z}_{i+1}+{h}_{1}{X}_{i}+{h}_{2}{X}_{i}{X}_{i+1}\right).$$

Ground states of (1) either belong to a topological quantum phase, a paramagnetic (PM) phase, or an antiferromagnetic phase depending on the model parameters {*h*_1_, *h*_2_}. *h*_1_ and *h*_2_ parametrize the strength of an external field and a nearest-neighbor Ising-type coupling in the model. {*X*_*i*_, *Y*_*i*_, *Z*_*i*_} are the Pauli operators acting on the spin at site *i*. We define ${Z}_{0}\equiv {Z}_{N+1}\equiv {X}_{N+1}\equiv {\mathbb{1}}$, which models a spin chain with open boundary conditions^41^.

**Fig. 1:**
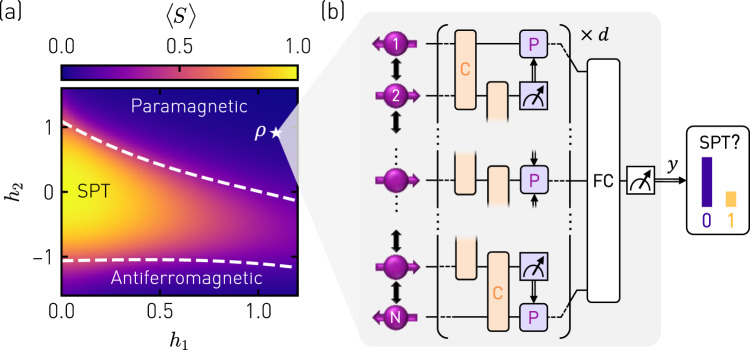
Concept of the quantum phase recognition experiment. **a** Phase diagram displaying the numerically calculated expectation value of the string order parameter $\left\langle {{{{{{{\mathcal{S}}}}}}}}\right\rangle =\left\langle {Z}_{1}{X}_{2}{X}_{4}{X}_{6}{Z}_{7}\right\rangle$ for ground states *ρ* of a cluster-Ising Hamiltonian (Eq. (1)) in the parameter space spanned by *h*_1_ and *h*_2_ for *N* = 7. The white dashed lines indicate the phase boundaries between the symmetry-protected topological (SPT) phase and the paramagnetic and antiferromagnetic phases, respectively. **b** An unknown state *ρ* drawn from the phase diagram in **a** is processed by a QCNN to recognize the phase to which it belongs. The QCNN consists of convolutional layers (C) decomposed into two-qubit gates (orange), of pooling layers (P) implemented as single-qubit operations conditioned on intermediate measurement outcomes (purple), a fully-connected circuit layer (FC), and the measurement of a single output qubit yielding outcome *y*.

In the thermodynamic limit, the bulk of the Hamiltonian *H* commutes with both even *P*_e_ = ∏_*i*_*X*_2*i*_ and odd *P*_o_ = ∏_*i*_*X*_2*i* + 1_ parity operators, and thus exhibits a ${{\mathbb{Z}}}_{2}\times {{\mathbb{Z}}}_{2}$ symmetry-protected topological quantum phase^43^, which falls into the same symmetry class as the *S* = 1 Haldane phase^44^. The SPT phase is distinguished from the paramagnetic and antiferromagnetic phase by a non-zero expectation value $\left\langle {{{{{{{\mathcal{S}}}}}}}}\right\rangle$ of the string order parameter^32^:

2 $${{{{{{{\mathcal{S}}}}}}}}={Z}_{1}{X}_{2}{X}_{4}...{X}_{N-3}{X}_{N-1}{Z}_{N},$$

which is defined for an odd number of spins *N*. Corresponding to the experimental situation in this work, we have computed $\left\langle {{{{{{{\mathcal{S}}}}}}}}\right\rangle$, shown in Fig. 1a, using exact diagonalization for a system of *N* = 7 spins. Due to the finite system size, we obtain smooth transitions across the phase boundaries (white dashed lines) determined from the maxima in the second derivative of the energy expectation value 〈*H*〉 with respect to *h*_2_^33^.

### Concept of the experiment

Conventionally, the phase to which an unknown quantum state *ρ* belongs is determined by measuring the expectation value of an order parameter ${{{{{{{\mathcal{S}}}}}}}}$, a process referred to as quantum phase recognition. However, when evaluating the expectation value $\left\langle {{{{{{{\mathcal{S}}}}}}}}\right\rangle$ by simultaneously, but individually measuring the qubits in their respective basis and by averaging the outcomes over multiple repetitions of the experiment, the sampling complexity increases close to the phase boundaries^11^. Furthermore, under realistic conditions the state *ρ*, which we prepare on the quantum hardware by executing a state-preparation circuit, might be subject to errors, reducing the value of $\langle {{{{{{{\mathcal{S}}}}}}}}\rangle$.

To overcome the aforementioned limitations, we perform quantum phase recognition by processing the trial states *ρ* with a QCNN. The structure of QCNNs, as recently proposed in Ref. ^11^, is inspired by classical convolutional neural networks widely used e.g., in image or speech recognition. A generic QCNN consists of alternating convolutional (C) and pooling (P) layers, followed by a fully-connected (FC) layer, as schematically shown in Fig. 1b. The combination of entangling gates applied between neighboring qubits in the convolutional layer, and single-qubit gates conditioned on the outcome of projective measurements in the pooling layer, reduces the number of qubits while retaining characteristic features of the input state vector. After repeating this procedure *d* times, a unitary operation in the FC layer maps the feature of interest onto a single output qubit. In general, QCNNs are parameterized and can be trained to identify specific features of interest.

In our particular case, the QCNN is designed to recognize string order and decide if the input state *ρ* belongs to the symmetry-protected topological phase or not. Instead of measuring the single string order parameters ${{{{{{{\mathcal{S}}}}}}}}$ as defined in Eq. (2), the output of the QCNN corresponds to the weighted sum of multiple string order parameters, the value of which is robust against local *X* and *Z*-type errors. The specific structure of the QCNN is inspired by the multiscale entanglement renormalization ansatz representation^45^ of the topological cluster state, which is the ground state of *H*(*h*_1_ = *h*_2_ = 0) = − ∑*Z*_*i*−1_*X*_*i*_*Z*_*i*+1_. In this case, each pair of convolutional and pooling layers maps a (perturbed) cluster state onto a cluster state of reduced system size, see Supplementary Note 5 for more details. Compared to the originally proposed QCNN^11^, we modify the FC layer to augment its tolerance to errors and use several gate identities to implement operations in the P and FC layers in classical processing wherever possible. We thereby drastically reduce the quantum gate count and enhance the performance of the QCNN under NISQ conditions.

For our experimental study carried out on a 7-qubit device, we combine two complementary elements. First, we prepare approximate ground states of the cluster-Ising Hamiltonian *H* by executing variational state preparation circuits. Second, we use those states as an input to the QCNN to demonstrate its capability to recognize the SPT phase and compare it to directly measuring 〈*S*〉.

### Variational state preparation

**Fig. 2:**
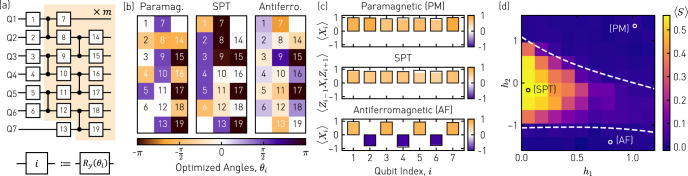
Variational ground state preparation. **a** Variational quantum circuit parametrized with 19 rotation angles ***θ*** used to prepare approximate ground states of the cluster-Ising Hamiltonian *H*. **b** Rotation angles *θ*_*i*_ found by an optimization algorithm on a conventional computer for three example states {*h*_1_, *h*_2_} in the paramagnetic (PM) {1.1, 1.4}, SPT {0.0, − 0.2}, and antiferromagnetic (AF) {0.8, − 1.4} phase. **c** Measured expectation values of the indicated operators (solid bars) along the qubit array in comparison to the simulated values (wire frames) for the three states prepared using the rotation angles in **b**. **d** Measured string order parameters $\left\langle {{{{{{{\mathcal{S}}}}}}}}\right\rangle$ for all prepared variational states vs. Hamiltonian parameters *h*_1_ and *h*_2_. Open circles indicate the three example states presented in **b** and **c**.

To prepare approximate ground states of the gapped one-dimensional Hamiltonian *H*^44^ for the entire parameter range {*h*_1_, *h*_2_} displayed in Fig. 1a, we use a low-depth, variational state preparation circuit *U*(***θ***) composed of three layers of single-qubit rotations *R*_*y*_(*θ*_*i*_) parametrized by 19 independent rotation angles *θ*_*i*_ and two layers of conditional-Z (CZ) gates interleaved with the single-qubit gates, see Fig. 2a^46^. We implement both types of gates directly on the quantum hardware, see Supplementary Note 4 in which we also provide a comparison to an alternative approach used in Ref. ^39^ to prepare ground states via an exact matrix product state representation of translationally invariant states.

To determine the variational parameters ***θ*** = {*θ*_1_, ..., *θ*_19_} corresponding to an approximate ground state of a specific *H*(*h*_1_, *h*_2_), we minimize the energy expectation value $\left\langle H\right\rangle$ in a conventional computer simulation with respect to the simulated output state $\left|{{{\boldsymbol{\theta}}}} \right\rangle =U({{{\boldsymbol{\theta}}}})\left|0\right\rangle$ by using a gradient based L-BFGS optimizer^47^. As an acceptance criterion for the convergence of the optimization algorithm, we compute the fidelity $F={|\langle {\psi }_{0}| {\theta }_{{{{{{{{\rm{opt}}}}}}}}}\rangle |}^{2}$ of the variational state $|{{{\boldsymbol{\theta}}}}_{{{{{{{{\rm{opt}}}}}}}}}\rangle$ with respect to the exact ground state $\left|{\psi }_{0}\right\rangle$, which, for *N* = 7, can be found using exact diagonalization. We repeat the optimization procedure with different initial values until *F* exceeds 81% solely being limited by the finite variational circuit depth of *m* = 1, see Supplementary Note 4. To make the state preparation circuit less susceptible to *T*_1_ errors, we then compute an equivalent set of rotational angles ${\tilde{{{\boldsymbol{\theta}}}}}_{{{{{{{{\rm{opt}}}}}}}}}$ yielding the same $U({\tilde{{{\boldsymbol{\theta}}}}}_{{{{{{{{\rm{opt}}}}}}}}})=U({{{\boldsymbol{\theta}}}}_{{{{{{{{\rm{opt}}}}}}}}})$^48^, but keeping the individual qubits preferentially in their respective ground state in the beginning of the state preparation sequence, see Supplementary Note 4 for details. This procedure avoids rotation angles close to *π* in the first layer of single-qubit rotations, which becomes apparent in the three examples shown in Fig. 2b by the absence of large rotation angles in the first column. The example state from the PM phase features rotation angles summing to ~ ±*π*/2 for each qubit individually. For the example state from the SPT phase, all qubits are initially rotated by an angle close to ±*π*/2, which, together with the subsequent layers of entangling CZ gates, results in an approximate cluster state^41^.

For the rotation angles ${\tilde{{{\boldsymbol{\theta}}}}}_{{{{{{{{\rm{opt}}}}}}}}}$ found in computer simulation, we execute the corresponding state preparation circuits on a 7-qubit superconducting quantum device featuring individual control and readout of all qubits, see Supplementary Note 1 for details. We realize single-qubit rotations by applying microwave pulses of controlled amplitude and phase and implement two-qubit CZ gates with flux pulses bringing the state $\left|11\right\rangle$ into resonance with the non-computational state $\left|20\right\rangle$^49,50^, where $|{n}_{i},{n}_{j}\rangle$ denote the states of the involved qubits in the Fock basis. To assure that all qubits are in their respective ground state at the beginning of each sequence, we perform a preselection readout and reject those measurement runs in which we found at least one of the qubits to be in the excited state, resulting in an overall acceptance probability of ~91%. We perform measurements in the *X* basis by prepending an *R*_*y*_(*π*/2) rotation to the respective qubits before performing standard dispersive readout in the *Z* basis. To mitigate the effect of readout infidelities on the order of 1.6% per qubit in averaged measurement observables, we multiply the vector of probabilities *p*_*i*_ of sampling the bitstring *x*_*i*_ by the inverse of the assignment probability matrix *M* to obtain ${\tilde{{{\boldsymbol{p}}}}}={M}^{-1}{{{\boldsymbol{p}}}}$, from which we evaluate expectation values, see Supplementary Note 2 for details.

To characterize our state preparation, we measure a set of local expectation values and compare the results with those obtained from Kraus operator simulations, which take qubit dissipation and dephasing, as well as measured CZ gate errors and nearest-neighbor residual ZZ coupling into account, see Fig. 2c. For states in the paramagnetic (antiferromagnetic) phase, we find, as expected, a non-zero magnetization $\langle {X}_{i}\rangle$ along the entire spin chain with equal (alternating) sign. The key signature of states in the SPT phase is the non-zero expectation value of Pauli strings $\left\langle {Z}_{i-1}{X}_{i}{Z}_{i+1}\right\rangle$. In all three cases, the average deviation between measured and simulated expectation values is below 5.8% and can likely be attributed to small additional coherent control errors, which we do not account for in the Kraus operator simulation.

**Fig. 3:**
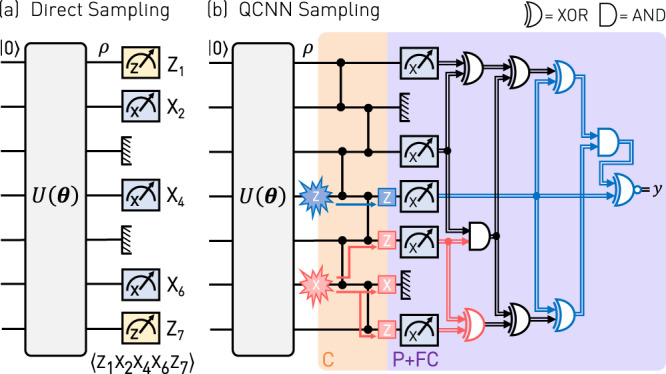
Quantum phase recognition circuits. **a** Quantum circuit for the case in which the qubits are measured in the indicated basis, directly after executing the state preparation circuit *U*(*θ*), to evaluate ${{{{{{{\mathcal{S}}}}}}}}={Z}_{1}{X}_{2}{X}_{4}{X}_{6}{Z}_{7}$. **b** QCNN circuit consisting of a convolutional layer (C) of CZ gates (orange), and a pooling (P) and fully-connected (FC) layer implemented as a measurement in the *X* basis with outcome ***x***, followed by a Boolean function *f*(***x***), here, represented by a logic circuit expressed in terms of AND and XOR gates (purple). An example of an *X* (*Z*) error occurring on qubit six (four) and its propagation through the QCNN is highlighted in red (blue).

To map out the full quantum phase diagram shown in Fig. 2d, we proceed with measuring the string order parameter $\left\langle {{{{{{{\mathcal{S}}}}}}}}\right\rangle$ for 10 × 10 combinations of {*h*_1_, *h*_2_} by directly sampling 32,768 times from the output of the state preparation circuit as indicated in Fig. 3a. We find the measured phase diagram to show all qualitative features of the exactly calculated one shown in Fig. 1a. The reduction in the overall contrast, which becomes apparent in the different amplitude scaling of $\langle {{{{{{{\mathcal{S}}}}}}}}\rangle$, stems from errors due to decoherence and two-qubit gate errors as confirmed by Kraus operator simulations, see Supplementary Note 4 for details. Most importantly, some of those errors can be tolerated when inferring the quantum phase of a prior unknown state by applying a QCNN algorithm rather than measuring $\left\langle {{{{{{{\mathcal{S}}}}}}}}\right\rangle$ directly, as we show in the following.

### Quantum phase recognition

Instead of measuring the expectations $\left\langle {{{{{{{\mathcal{S}}}}}}}}\right\rangle$ directly after state preparation, we now employ the QCNN to detect the SPT phase. For this purpose, we process the prepared quantum states by the QCNN depicted in Fig. 3b and evaluate the expectation value 2〈*y*〉 − 1 of the single output bit *y*, which corresponds to measuring the expectation value of a multiscale string order parameter ${{{{{{{{\mathcal{S}}}}}}}}}_{{{{{{{{\rm{M}}}}}}}}}$ consisting of a weighted sum of Pauli strings, see Eq. (14) in Supplementary Note 5 for an explicit expression. Measuring the QCNN output ${{{{{{{{\mathcal{S}}}}}}}}}_{{{{{{{{\rm{M}}}}}}}}}=2y-1$ instead of ${{{{{{{\mathcal{S}}}}}}}}$ is advantageous because weakly perturbed cluster states still yield ${{{{{{{{\mathcal{S}}}}}}}}}_{{{{{{{{\rm{M}}}}}}}}}=+1$ while the same states have a finite probability to yield ${{{{{{{\mathcal{S}}}}}}}}=-1$ which reduces the fidelity of the respective expectation value. Due to this property, ${{{{{{{{\mathcal{S}}}}}}}}}_{{{{{{{{\rm{M}}}}}}}}}$ attains a step-like behavior at phase boundaries for large system sizes leading to a reduced sampling complexity^11^. Using the QCNN allows us to measure all the individual strings in ${{{{{{{{\mathcal{S}}}}}}}}}_{{{{{{{{\rm{M}}}}}}}}}$ simultaneously, thereby reducing the number of measurement terms compared to a direct sampling of all Pauli strings, by an amount which scales exponentially with the QCNN system size *N*. For the system size *N* = 7 considered in our experiment the number of individual strings in ${{{{{{{{\mathcal{S}}}}}}}}}_{{{{{{{{\rm{M}}}}}}}}}$ is 10, see Supplementary Note 5 for details.

We construct the QCNN by making two modifications to the quantum circuit proposed in Ref. ^11^. First, we perform the pooling and fully-connected layers as a Boolean function *f*(***x***) after having performed a projective measurement. This approach requires only a constant depth quantum circuit irrespective of the qubit number, which greatly reduces the total quantum gate count in comparison to preceding QCNN circuits while yielding the same output value. We explicitly show in Supplementary Note 5 that the modified QCNN circuit is fully equivalent to the original circuit in which all operations are implemented with quantum gates. While this modification is applicable to the exact QCNN circuit optimized for our particular model system, the implementation of a generic parametrized QCNN may not allow for a similar reduction of quantum gates. Second, we extend the fully-connected layer to map the measured bitstring ***x*** = (*x*_1_, *x*_3_, *x*_4_, *x*_5_, *x*_7_) onto a single output bit *y*, such that it not only tolerates *X* errors, but also *Z* errors, provided the errors are sufficiently sparse. For example, an *X* error occurring on qubit six and a *Z* error on qubit four prior to the convolutional layer invert bits *x*_5_, *x*_7_ and *x*_4_, which is corrected by the function *f*(***x***), see the red and blue colored paths in Fig. 3b. With this modification, the number of measurements that would be needed to determine $\langle {{{{{{{{\mathcal{S}}}}}}}}}_{{{{{{{{\rm{M}}}}}}}}}\rangle$ without employing the QCNN is also much larger than in previously proposed versions^11^, for which all the involved Pauli strings can be obtained from measuring in only two different bases, see Supplementary Note 5. The constant-depth quantum circuit plays a crucial role in the QCNN as it allows us to simultaneously sample all quasi-local observables *Z*_*i−1*_*X*_*i*_*Z*_*i*+1_. The samples contain information about the long-range correlations between these quasi-local observables, which enables us to efficiently determine $\langle {{{{{{{{\mathcal{S}}}}}}}}}_{{{{{{{{\rm{M}}}}}}}}}\rangle$ using classical processing.

**Fig. 4:**
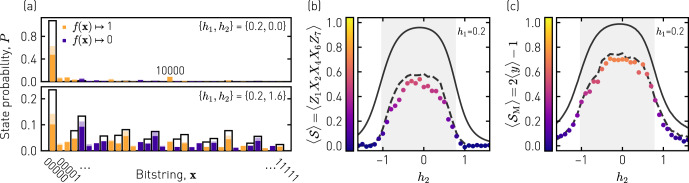
Performance of the QCNN. **a** Probability to sample bitstrings *x* after having applied the convolutional layer (compare Fig. 3b) for the two indicated Hamiltonian parameter sets {*h*_1_, *h*_2_}. Bitstrings mapped onto 1 (0) by the function *f*(***x***) are colored in orange (purple) and the expected probabilities from a Kraus operator simulation in the corresponding light color, whereas the ideal values are depicted as black wire frames. **b** Expectation value $\left\langle {{{{{{{\mathcal{S}}}}}}}}\right\rangle =\left\langle {Z}_{1}{X}_{2}{X}_{4}{X}_{6}{Z}_{7}\right\rangle$ measured directly after variational state preparation (compare Fig. 3a) vs. *h*_2_ for fixed *h*_1_ = 0.2, in comparison to the ideal values (solid line) and simulated values taking decoherence into account (dashed line). The SPT phase is indicated in light gray. **c** Expectation value $2\left\langle y\right\rangle -1$ measured after applying the QCNN for the same parameters as in **b** compared to values extracted from a Kraus operator simulation of the QCNN circuit (dashed line) and the ideal value (solid line).

To investigate the QCNN’s tolerance to errors in more detail, we sample *x* after having performed the convolutional layer for two different ground states chosen from the SPT and PM phase, respectively, and obtain the probability distributions shown in Fig. 4a. For the state in the SPT phase (top panel), we find a high probability of 0.47 to sample (00000), which is expected because the ideal cluster state corresponding to the ground state of *H*(*h*_1_ = *h*_2_ = 0) is mapped onto $\left|{+}{+}{+}{+}{+}\right\rangle$ by the disentangling CZ gates of the quantum convolutional layer. However, due to the non-zero value of *h*_1_ = 0.2 and the presence of noise in the quantum circuit, we also measure other bitstrings with non-zero probability, most notably (10000). Most importantly, a large fraction of those bitstrings is correctly mapped onto *y* = 1 by the function *f*, thereby counteracting a quantum phase misclassification in those cases. For the paramagnetic example (bottom panel), we find the sampled bitstrings to be more uniformly distributed and, correspondingly, *y* to result equally likely in 0 or 1. In both examples, we find the measured probability distributions to be in good agreement with the simulated ones, taking decoherence, two-qubit gate imperfections and readout errors into account.

We finally quantify the performance of the QCNN in correctly identifying the SPT phase by comparing the value 2〈*y*〉 − 1 obtained from the QCNN to the value $\left\langle {{{{{{{\mathcal{S}}}}}}}}\right\rangle$ obtained from direct sampling, see Fig. 4b, c. In particular, we determine both quantities across the phase boundaries separating the SPT phase (light gray) from the paramagnetic and antiferromagnetic phases, respectively, by varying *h*_2_ for constant *h*_1_ = 0.2. In both cases the measured expectation values (dots), which we obtain from 32,768 individual samples, approach zero for the paramagnetic and antiferromagnetic phases and take a non-zero value reaching 0.70 in the SPT phase. Compared to the ideal values (solid lines) and as a result of error events, the overall fidelity is reduced - an effect which is well-explained by Kraus operator simulations of the respective quantum circuits (dashed lines), which also identify two-qubit gate imperfections as the most prominent error contributor, see Supplementary Note 4 for details on the simulations. Most importantly, we find the average difference of the measured QCNN output values 2〈*y*〉 − 1 from the ideal curve to be 0.23, while the average difference of the directly measured values of $\left\langle {{{{{{{\mathcal{S}}}}}}}}\right\rangle$ is 0.34. This enhancement of performance provides clear evidence for the robustness of the QCNN against errors.

## Discussion

By implementing a QCNN on a superconducting quantum processor, we have demonstrated its capability to efficiently recognize quantum phases. With further advances in qubit number and circuit depth, we expect QCNNs to become an important diagnostic tool to characterize output states of NISQ devices, which are increasingly challenging to analyze with classical computing. Such applications will benefit from the predicted increased sampling complexity at phase boundaries^11^, at which the QCNN enhances the distinguishing power for assigning states to different quantum phases. While this enhancement is not yet accessible for the system size considered here, larger systems will be able to benefit from it, see Supplementary Note 5. Additionally, the scaling advantage can be understood by expressing the output of a QCNN by an equivalent weighted sum of string order parameters, the number of which scales exponentially with *N*. The QCNN thus allows one to simultaneously measure the sum of all those terms. An interesting direction to be explored in future work includes the trainability of parameterized QCNNs. This also becomes relevant in the context of using QCNNs to learn optimal strategies for quantum error correction.

## Supplementary information

Supplementary Information

## Data Availability

The authors declare that the data supporting the findings of this letter and corresponding Supplementary Information file are available online at the ETH Zurich repository for research data https://doi.org/10.3929/ethz-b-000530297.

## References

[1] Arute F (2019). Quantum supremacy using a programmable superconducting processor. Nature.

[2] Zhong H (2020). Quantum computational advantage using photons. Science.

[3] Egan, L. et al. Fault-tolerant operation of a quantum error-correction code. https://arxiv.org/abs/2009.11482 (2020).

[4] Andersen CK (2020). Repeated quantum error detection in a surface code. Nat. Phys..

[5] Preskill J (2018). Quantum computing in the NISQ era and beyond. Quantum.

[6] Montanaro A (2016). Quantum algorithms: an overview. npj Quantum Inf..

[7] Feynman RP (1982). Simulating physics with computers. Int. J. Theor. Phys..

[8] Cao Y (2019). Quantum chemistry in the age of quantum computing. Chem. Rev..

[9] Biamonte J (2017). Quantum machine learning. Nature.

[10] Farhi, E. & Neven, H. Classification with quantum neural networks on near term processors. https://arxiv.org/abs/1802.06002 (2018).

[11] Cong I, Choi S, Lukin MD (2019). Quantum convolutional neural networks. Nat. Phys..

[12] Beer K (2020). Training deep quantum neural networks. Nat. Commun..

[13] Seber, G. A. F. & Lee, A. J. *Linear Regression Analysis* 2nd edn (Wiley, 2003).

[14] Harrow AW, Hassidim A, Lloyd S (2009). Quantum algorithm for linear systems of equations. Phys. Rev. Lett..

[15] Scherer A (2017). Concrete resource analysis of the quantum linear-system algorithm used to compute the electromagnetic scattering cross section of a 2D target. Quantum Inf. Process..

[16] Aaronson S (2015). Read the fine print. Nat. Phys..

[17] Havlicek V (2019). Supervised learning with quantum-enhanced feature spaces. Nature.

[18] Schuld M, Killoran N (2019). Quantum machine learning in feature hilbert spaces. Phys. Rev. Lett..

[19] Jaderberg, B. et al. Quantum self-supervised learning. https://arxiv.org/abs/2103.14653 (2021).

[20] Grant E (2018). Hierarchical quantum classifiers. npj Quantum Inf..

[21] Lloyd S, Mohseni M, Rebentrost P (2014). Quantum principal component analysis. Nat. Phys..

[22] Zhang X-M (2021). Generic detection-based error mitigation using quantum autoencoders. Phys. Rev. A.

[23] Bondarenko D, Feldmann P (2020). Quantum autoencoders to denoise quantum data. Phys. Rev. Lett..

[24] Romero J, Olson JP, Aspuru-Guzik A (2017). Quantum autoencoders for efficient compression of quantum data. Quantum Sci. Technol..

[25] Gentile AA (2021). Learning models of quantum systems from experiments. Nat. Phys..

[26] Wiebe N, Granade C, Ferrie C, Cory DG (2014). Hamiltonian learning and certification using quantum resources. Phys. Rev. Lett..

[27] Kottmann K, Metz F, Fraxanet J, Baldelli N (2021). Variational quantum anomaly detection: unsupervised mapping of phase diagrams on a physical quantum computer. Phys. Rev. Research.

[28] Ghosh S, Opala A, Matuszewski M, Paterek T, Liew TCH (2019). Quantum reservoir processing. npj Quantum Inf..

[29] Ghosh S, Nakajima K, Krisnanda T, Fujii K, Liew TCH (2021). Quantum neuromorphic computing with reservoir computing networks. Adv. Quantum Technol..

[30] Pollmann F, Turner AM, Berg E, Oshikawa M (2010). Entanglement spectrum of a topological phase in one dimension. Phys. Rev. B..

[31] Chen Y-F, Gu Z-C, Xiao-Gang W (2011). Classification of gapped symmetric phases in one-dimensional spin systems. Phys. Rev. B..

[32] Pollmann F, Turner AM (2012). Detection of symmetry-protected topological phases in one dimension. Phys. Rev. B..

[33] Sachdev S (2001). Quantum Phase Transitions.

[34] Wang ZF (2016). Topological edge states in a high-temperature superconductor FeSe/SrTiO_3_(001) film. Nat. Mat..

[35] Carrasquilla J, Melko RG (2017). Machine learning phases of matter. Nat. Phys..

[36] Rodriguez-Nieva JF, Scheurer MS (2019). Identifying topological order through unsupervised machine learning. Nat. Phys..

[37] Greplova E (2020). Unsupervised identification of topological phase transitions using predictive models. New J. Phys..

[38] Lian W (2019). Machine learning topological phases with a solid-state quantum simulator. Phys. Rev. Lett..

[39] Smith A, Jobst B, Green AG, Pollmann F (2022). Crossing a topological phase transition with a quantum computer. Phys. Rev. Res..

[40] Satzinger KJ (2021). Realizing topologically ordered states on a quantum processor. Science.

[41] Azses, D. et al. Identification of symmetry-protected topological states on noisy quantum computers. *Phys. Rev. Lett.* **125**, 120502 (2020). 10.1103/PhysRevLett.125.120502 33016759

[42] Smacchia P (2011). Statistical mechanics of the cluster Ising model. Phys. Rev. A..

[43] Verresen R, Moessner R, Pollmann F (2017). One-dimensional symmetry protected topological phases and their transition. Phys. Rev. B..

[44] Haldane FDM (1983). Nonlinear field theory of large-spin heisenberg antiferromagnets: semiclassically quantized solitons of the one-dimensional easy-axis neel state. Phys. Rev. Lett..

[45] Vidal G (2008). Class of quantum many-body states that can be efficiently simulated. Phys. Rev. Lett..

[46] Bravo-Prieto C, Lumbreras-Zarapico J, Tagliacozzo L, Latorre JI (2020). Optimal fermion-to-qubit mapping via ternary trees with applications to reduced quantum states learning. Quantum.

[47] Byrd RH, Lu P, Nocedal J, Zhu C (1995). A limited memory algorithm for bound constrained optimization. SIAM J. Sci. Comput..

[48] Fontana, E., Cerezo, M., Arrasmith, A., Rungger, I. & Coles, P. J. Optimizing parametrized quantum circuits via noise-induced breaking of symmetries. https://arxiv.org/abs/2011.08763 (2020).

[49] Strauch FW (2003). Quantum logic gates for coupled superconducting phase qubits. Phys. Rev. Lett..

[50] DiCarlo L (2009). Demonstration of two-qubit algorithms with a superconducting quantum processor. Nature.

