# Supplementary material for: Realizing quantum convolutional neural networks on a superconducting quantum processor to recognize quantum phases
Source: Nat Commun. 2022 Jul 16;13:4144. doi: 10.1038/s41467-022-31679-5 (PMC9288436; doi:10.1038/s41467-022-31679-5)
Supplement: Supplementary file 1 — Supplementary Information [file 41467_2022_31679_MOESM1_ESM.pdf]

Supplementary Information to Realizing Quantum Convolutional Neural Networks on a Superconducting Quantum Processor to Recognize Quantum Phases

Johannes Herrmann,[1,*] Sergi Masot Llima,[1] Ants Remm,[1] Petr Zapletal,[2] Nathan A. McMahon,[2] Colin Scarato,[1] François Swiadek,[1] Christian Kraglund Andersen,[1] Christoph Hellings,[1] Sebastian Krinner,[1] Nathan Lacroix,[1] Stefania Lazar,[1] Michael Kerschbaum,[1] Dante Colao Zanuz,[1] Graham J. Norris,[1] Michael J. Hartmann,[2] Andreas Wallraff,[1,3] and Christopher Eichler[1,†]

[1]*Department of Physics, ETH Zurich, CH-8093 Zurich, Switzerland*
[2]*Department of Physics, Friedrich-Alexander University Erlangen-Nürnberg (FAU), Erlangen, Germany*
[3]*Quantum Center, ETH Zurich, CH-8093 Zurich, Switzerland*

(Dated: May 24, 2022)

SUPPLEMENTARY NOTES

Supplementary Note 1: Experimental Setup

Device fabrication – We fabricated the 7-qubit quantum processor, shown in Supplementary Fig. 2, by sputtering a Niobium thin film onto a high-resistivity intrinsic Silicon substrate in a process similar to the one described in Ref. [1]. After patterning the Niobium base layer using photolithography and reactive-ion etching, we fabricate airbridges to establish well-connected ground planes and to enable crossings of signal lines. We fabricate Josephson junctions by shadow evaporation of aluminum through a resist mask defined by electron-beam lithography.

Device Parameters – We extract the qubit and readout circuit parameters, summarized in Supplementary Table 1, using standard spectroscopy and time-domain measurements. We extract the quantum measurement efficiencies using the methods described in Refs. [2, 3].

Wiring and instrumentation – We install the device at the base plate of a cryogenic measurement setup (13 mK) and connect it to the control and measurement electronics as shown in Supplementary Fig. 1. We control the individual qubit frequencies by threading a magnetic flux through the superconducting quantum interference device (SQUID) loop via a current applied to an inductively coupled flux control line. The flux control signal is composed of a constant offset superimposed with pulses controlled on the nanosecond timescale. The constant offset, which we generate using a voltage source (SRS SIM928) and a 1 kΩ bias resistor in series, tunes the qubit to its idle frequency. Fast pulses, which we generate using an arbitrary waveform generator (Tektronix AWG5014C), are used to activate two-qubit gates (see Supplementary Note 2 for details). We combine both signals at room-temperature using a bias-tee (Mini-Circuits ZFBT-4R2GW+) with a primary timeconstant of $\sim$18 μs to filter out low-frequency noise present at the output of the AWG. We achieve XY-control of all seven qubits by up-converting the in-phase and quadrature components of an intermediate frequency signal with analog IQ-mixer modules (Zurich Instruments HDIQ). Qubit drive pulses are provided by two 8-channel AWGs with a sample rate of 2.4 GSa/s (Zurich Instruments HDAWG).

We perform frequency-multiplexed qubit readout using two FPGA-based control systems with a sampling rate of 1.8 GSa/s (Zurich Instruments UHFQA). The multi-chromatic readout pulse is upconverted to the readout resonator frequency band and routed through a highly attenuated RF line to the readout line on the chip. At the output, each one of the two frequency-multiplexed readout lines is connected to an amplification chain consisting of a traveling wave parametric amplifier (TWPA) [4] at base, a cryogenic high-electron mobility transistor (HEMT) at 4 K, and additional low-noise amplifiers at room temperature (RT-AMP).

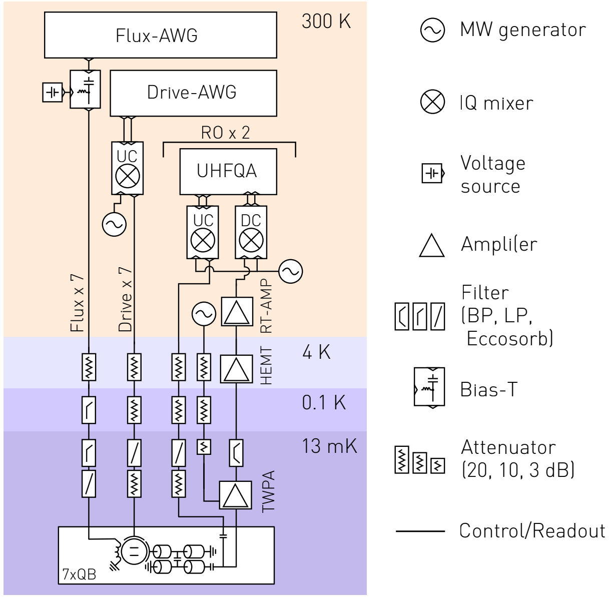

Supplementary Figure 1. Schematic of the control electronics and wiring setup. For details see main text.

* johannes.herrmann@phys.ethz.ch
† eichlerc@phys.ethz.ch

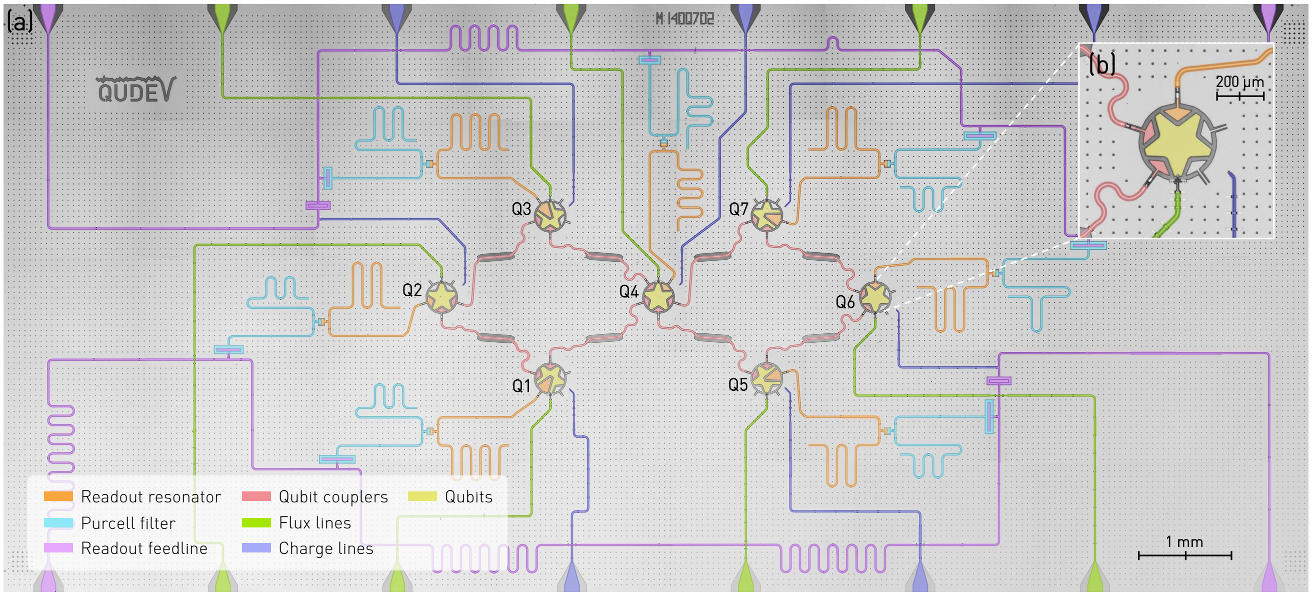

Supplementary Figure 2. (a) False-color micrograph of the 7-qubit quantum processor with individual elements specified in the legend on the bottom left. (b) Enlarged view of the transmon qubit Q6 and its connecting lines.

	Q1	Q2	Q3	Q4	Q5	Q6	Q7
Qubit idle frequency, $\omega_{\mathrm{q}}/2\pi$ (GHz)	4.183	5.949	4.453	5.881	4.522	6.071	4.200
Qubit anharmonicity, $\alpha_{\mathrm{q}}/2\pi$ (MHz)	-181	-170	-177	-173	-178	-166	-179
Lifetime, T_1 (μs)	38.1	13.5	19.8	13.7	19.8	16.4	15.6
Ramsey decay time, T_2^* (μs)	20.7	12.9	14.3	10.1	13.1	19.1	8.2
Echo decay time, T_2^{e} (μs)	33.2	14.1	16.7	10.4	18.5	22.1	20.5
Readout resonator frequency, $\omega_{\mathrm{r}}/2\pi$ (GHz)	6.668	7.089	6.603	7.213	6.904	6.994	6.812
Readout linewidth, $\kappa_{\mathrm{eff}}/2\pi$ (MHz)	11	7	8	12	10	11	9
Dispersive Shift, $\chi/2\pi$ (MHz)	-3.5	-3.6	-3.0	-1.9	-2.0	-2.4	-2.5
Thermal population, P_{th}(%)	3.1	0.7	2.7	0.7	1.3	1.3	1.6
Individual readout assignment prob. (%)	99.2	98.7	98.5	97.7	99.4	99.2	98.4
Multiplexed readout assignment prob. (%)	99.1	98.2	98.2	97.4	96.4	97.7	98.2
Measurement efficiency, η (%)	34.0	31.3	15.4	15.9	23.4	25.3	14.7

Supplementary Table 1. Measured parameters of the seven qubits.

The amplified signals are finally downconverted to an intermediate frequency band, digitized and integrated by the weighted integration units of the UHFQAs.

Supplementary Note 2: Control and Readout

Single-qubit gates – To achieve XY-control we generate pulses having a carrier frequency resonant with the qubit transition frequency and an envelope following a DRAG pulse parametrization to reduce leakage into non-computational states [5]. We choose a pulse width of $\sigma = 10\,$ns and truncate to a pulse length of $\tau = 5\sigma = 50\,$ns. To implement rotations $R_y(\theta_i)$ with a continuously adjustable target angle $\theta_i \in]-\pi, +\pi]$, we linearly scale the calibrated π-pulse amplitude A_π to $A_\theta = A_\pi \theta/\pi$.

Two-qubit gates – We perform two-qubit controlled-Z (CZ) gates by harnessing the in-situ tuneability of the transition frequencies of the qubits. Our choice of idle frequencies places the qubits in two main frequency bands, where neighboring qubits alternate between upper and lower sweet spot, see dashed lines in Supplementary Fig. 3(a). The large detuning of those frequency bands by approximately 1.7 GHz keeps residual ZZ coupling during idle times [6, 7] below 15 kHz for all qubit pairs, see Supplementary Note 3 for details. In addition, biasing the qubits to one of their sweet spots reduces the susceptibility to flux noise. To activate a two-qubit CZ gate, we tune both participating qubits to an intermediate interaction frequency such that the $|11\rangle$ and the non-computational $|20\rangle$ state become resonant [8, 9]. By choosing an intermediate interaction frequency, we

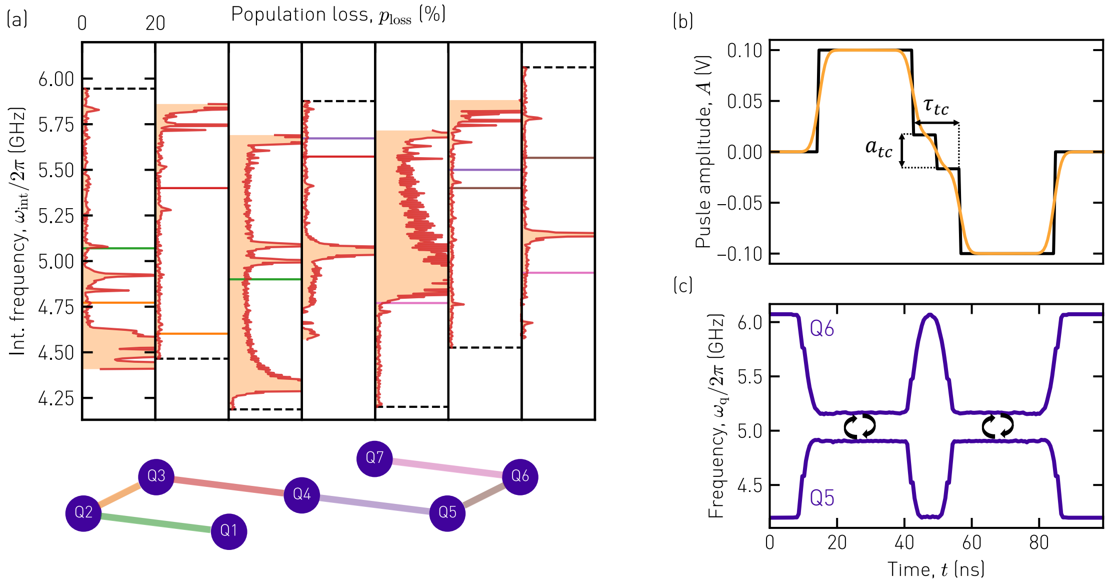

Supplementary Figure 3. **Two-qubit gate implementation.** (a) Measured population loss for all qubits when being tuned from the idle frequency (dashed horizontal line) to the frequency ω_{int} for a duration of 100 ns. Colored lines indicate the chosen interaction frequencies during two-qubit gates for the respective qubit pair. (b) Parametrization of the net-zero transition-controlled flux pulse, with an adjustable transition amplitude a_{tc} in the transition part of length τ_{tc} between the two halves of the net-zero pulse before (black) and after (orange) applying an additional Gaussian filtering with $\sigma = 0.5$ ns. (c) Measured time-dependent frequencies of qubits Q5 and Q6 in response to the flux pulses shown in (b) with predistortion applied. Frequencies at which the $|11\rangle$ and $|20\rangle$ states interact resonantly are indicated by round arrows.

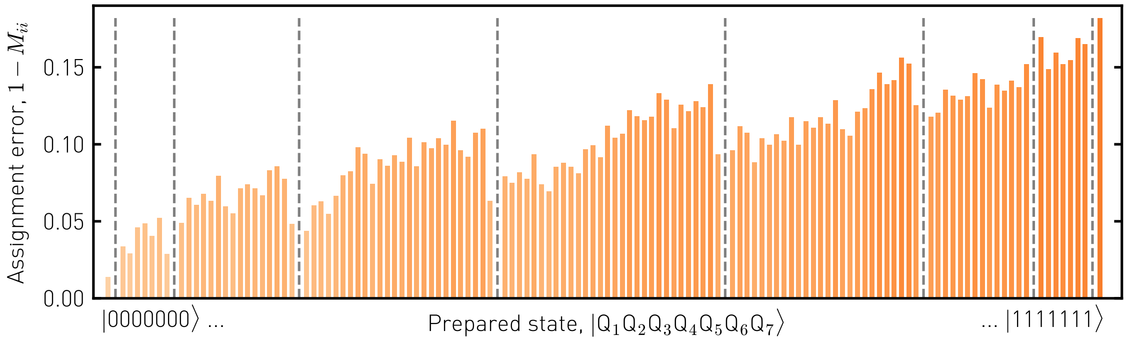

Supplementary Figure 4. **Multiplexed qubit readout.** Assignment error $1 - M_{ii}$, for the simultaneous single-shot readout of all seven qubits. The prepared states are sorted by the number of qubit excitations (separation by the dashed gray lines) and arranged in binary ascending order within their subdivision.

avoid frequency collisions with neighboring qubits, which are not supposed to participate in the gate. The exact choice of interaction frequencies, see colored lines in Supplementary Fig. 3(a), is governed by the avoidance of frequency-dependent population loss, which is most likely caused by the interaction with two-level systems residing at the material interfaces and inside the tunnel junctions [10, 11]. We characterize the population loss for each qubit individually, by measuring the remaining population of an initially prepared excited state after a 100 ns long rectangular flux pulse smoothed with a Gaussian filter of width $\sigma = 0.5$ ns, which tunes the respective qubit to the variable interaction frequency ω_{int}, see Supplementary Fig. 3(a).

The flux pulses activating the two-qubit gate interaction have a net-zero transition-controlled (NZTC) pulse shape, as depicted in Supplementary Fig. 3(b), consisting of a NZ pulse to assure flux pulse repeatability and an additional adjustable amplitude step to provide control over the transition part [12, 13]. We parametrize the transition part of the pulse with an individual length τ_{tc} and amplitude a_{tc}. We keep τ_{tc} fixed to 2.5 ns and use the amplitude a_{tc} to calibrate the sudden phase jump between the interacting $|11\rangle$ and $|20\rangle$ states when the NZTC pulse

changes sign [13]. We calibrate the total length and the main amplitude of the flux pulse, such that a conditional phase of π and full population recovery from the $|20\rangle$ state is achieved. The average flux pulse length is 71 ns. To suppress SWAP errors in the single-excitation manifold, we apply a Gaussian filter of width $\sigma = 0.5$ ns to all flux pulses. To preclude a possible overlap with preceding and subsequent pulses we add 20 ns-long buffer periods before and after each flux pulse.

Due to the high-pass filtering characteristic of the microwave bias-tee and imperfections in the impedance matching of the flux control line, the flux pulses are subject to distortions on small and long timescales, which we correct for by applying finite impulse response (FIR) and infinite impulse response (IIR) filters to the programmed waveforms. We extract the corresponding IIR filter coefficients from flux pulse scope measurements of the qubit frequency over timescales ranging from 50 ns to 20 μs. We correct for pulse distortions on the nanosecond timescale by extracting a set of FIR filter coefficients for each qubit using the cryoscope method described in Ref. [14]. To verify accurate pulse control, we measure the time-dependent qubit frequency in response to the NZTC flux pulses, exemplary shown for the gate between Q5 and Q6 in Supplementary Fig. 3(c).

Multiplexed single-shot readout – To readout the state of all qubits simultaneously, we perform frequency-multiplexed qubit readout as described in Ref. [3] using 600 ns long Gaussian-filtered square pulses. We multiply the digitized readout signal with a set of optimal integration weights, integrate for a period of 650 ns and threshold the resulting value to discriminate between the two qubit states. To evaluate the performance of the qubit readout, we take $n = 10{,}000$ single-shot measurements for each of the 128 possible qubit basis state combinations. Based on the outcome of an additional preselection readout and for each prepared basis state i, we select only those $n_0 \approx 0.91n$ of the shots for further analysis, for which all qubits were initially found in the ground state. For each basis state i, we determine the frequency $f(j|i)$ with which we assigned the state label j to obtain an estimate of the assignment probability matrix $M_{ji} \equiv f(j|i)/n_0$. As shown in Supplementary Fig. 4, the assignment error $1 - M_{ii}$ increases with excitation number due to qubit relaxation during the readout. As described in the main text, we account for readout errors by multiplying any measured probability distribution p_i with the inverse of M before evaluating expectation values of observables. When qubits are read out individually, e.g. for standard calibration and characterization measurements, we achieve the assignment fidelities stated in Supplementary Table 1.

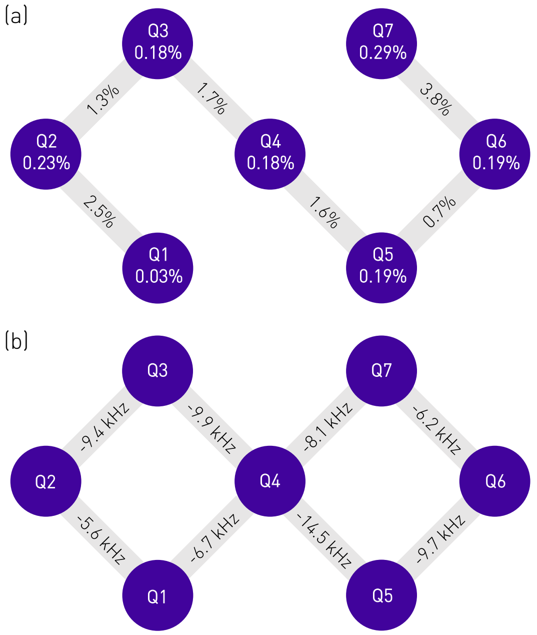

Supplementary Figure 5. **Gate performance.** (a) Single and two-qubit gate infidelities, extracted from randomized benchmarking and quantum process tomography, respectively. (b) Measured residual ZZ coupling strength α_{zz} between neighboring qubits at idle frequencies.

Supplementary Note 3: Gate characterization

Single-qubit gate performance – We characterize the single-qubit gate performance by individual randomized benchmarking and find an average fidelity of 99.82 % per Clifford, see Supplementary Fig. 5(a). To determine the residual ZZ coupling strength α_{zz}, we measure the shift in qubit frequency using a Ramsey experiment, induced by an excitation of the neighboring qubit [6]. We find values well below 15 kHz, see Supplementary Fig. 5(b).

Two-qubit gate performance – We calibrate and characterize six CZ gates on the quantum device, such that a qubit connectivity in a 1D chain is established, and use quantum process tomography to evaluate the gate performance by determining the corresponding process $\mathcal{E}(\rho) = \sum_{\alpha,\beta=1}^{16} \chi_{\alpha\beta} E_\alpha \rho E_\beta^\dagger$, where $E_\alpha \in \{I, X, Y, Z\}^{\otimes 2}$. For that purpose, we prepare $R_i|0\rangle$

	CZ_{12}	CZ_{23}	CZ_{34}	CZ_{45}	CZ_{56}	CZ_{67}
Estimated infidelity (%)	2.4	0.5	0.6	0.6	0.6	2.1
Measured infidelity (%)	2.5	1.3	1.7	1.6	0.7	3.8

Supplementary Table 2. Estimated bound for CZ gate infidelities calculated from the measured population loss at the gate interaction frequency in comparison to the measured gate infidelities obtained from quantum process tomography.

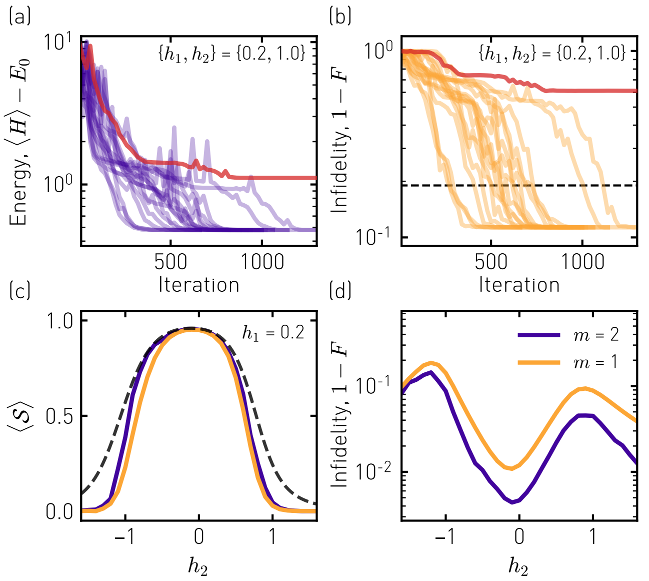

Supplementary Figure 6. **Variational circuit optimization.** (a) Ground state energy $\langle H \rangle$ as a function of the optimization step for $n = 20$ different runs from randomly chosen starting conditions. The energy is stated as a difference to the ideal ground state energy $\langle H \rangle$ for the specified Hamiltonian parameters $\{h_1, h_2\}$. (b) Infidelity of the quantum state as a function of the optimization step with the acceptance threshold of 10 % being marked with a dashed black line. The red curves in (a) and (b) depict an unsuccessful optimization run into a local minimum. Value of $\langle S \rangle$ in (c) and infidelity $1 - F$ in (d) for a fixed $h_1 = 0.2$ vs. h_2 for different variational circuit depths m compared to the ideal value (dashed line).

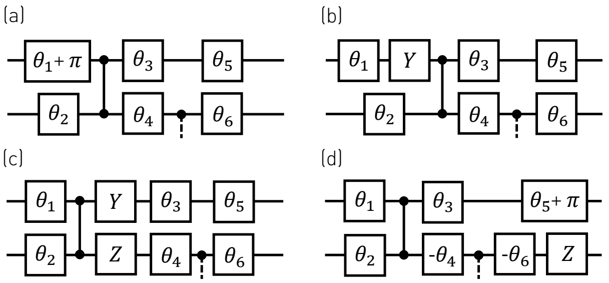

Supplementary Figure 7. **Exploiting ansatz symmetries.** Sequence of equivalent quantum circuits showing how to eliminate a rotation angle $\theta_1 + \pi$ close to π in the first circuit layer by adjusting the rotation angles in the second and third layer.

with $R_i \in \{I, R_x(\pi/2), R_y(\pi/2), R_x(\pi)\}^{\otimes 2}$, apply the CZ gate and perform quantum state tomography for each of the 16 different R_i. To reduce the effect of state preparation errors, we condition the data analysis on having detected the qubits in the ground state initially. To account for readout errors, we multiply the obtained averaged state probabilities $\boldsymbol{p}$ by the inverse of the measured readout assignment probability matrix M to obtain $\tilde{\boldsymbol{p}} = M^{-1}\boldsymbol{p}$. Based on the probability distributions $\tilde{\boldsymbol{p}}$, we reconstruct the most likely density matrix ρ_i using a maximum-likelihood procedure. Based on those density matrices, we compute the process matrix χ following the procedure in Ref. [15]. We compute the respective gate infidelities $1 - F = 1 - \mathrm{Tr}\,(\chi_{\mathrm{cz}}\chi)$ listed in Supplementary Fig. 5(a) by comparing χ to the ideal CZ process matrix χ_{cz} and use the experimentally obtained process matrices for the Kraus operator simulation in Supplementary Note 4.

To determine the contribution of population loss to the two-qubit gate infidelity, we measure the probabilities $p_{\mathrm{h},|1\rangle}$ and $p_{\mathrm{l},|1\rangle}$ for an initial excited state to decay during the flux pulse for both the high (h) and low (l) frequency qubit at the gate interaction frequency, see Supplementary Fig. 3(a). To account for a possible decay event of the high frequency qubit from the second excited state during the gate, we also measure the population loss $p_{\mathrm{h},|2\rangle}$ at the corresponding interaction frequency. We calculate the expected gate infidelity by modeling the dynamics of our two-qubit gate of length τ with the following master equation

$$\begin{aligned}\dot{\rho} = &- \mathrm{i}[H_q, \rho] + \frac{p_{\mathrm{l},|1\rangle}}{\tau} L_{|0\rangle\langle 1|}[\rho] \\ &+ \frac{p_{\mathrm{h},|1\rangle}}{\tau} L_{|0\rangle\langle 1|}[\rho] + \frac{p_{\mathrm{h},|2\rangle}}{\tau} L_{|1\rangle\langle 2|}[\rho]\end{aligned} \tag{1}$$

where

$$L_a[\rho] = a\rho a^\dagger - \frac{1}{2}(a^\dagger a\rho + \rho a^\dagger a) \tag{2}$$

are Lindblad operators modelling decay and

$$H_q = \frac{\pi}{\tau}\left(|11\rangle\langle 02| + |02\rangle\langle 11|\right) \tag{3}$$

is the Hamiltonian describing the interaction in the second excitation manifold. We compute the corresponding process matrix χ by evolving (1) for the two-qubit gate duration τ, determine the expected gate infidelity $1 - F = 1 - \mathrm{Tr}\,(\chi_{\mathrm{cz}}\chi)$ and summarize the obtained values in Supplementary Table 2. We find that the gates CZ_{12} and CZ_{67} are most strongly affected by population loss during the gate operation, which is consistent with the measured infidelities, see Supplementary Table 2.

Supplementary Note 4: Methods

Variational circuit optimization – As explained in the main text, we simulate the variational state preparation circuit, shown in Fig. 2(a), to minimize the expectation of $\langle H \rangle$ using the L-BFGS optimizer from the Qiskit Python package [16]. As depicted in Supplementary Fig. 6(a) and (b), the circuit optimization typically converges after 500 to 1,000 iterations achieving an average energy accuracy $\langle H \rangle - E_0 \sim 0.4$ and average state infidelity $1 - F \sim 6.9$ }, where E_0 is the ground state energy

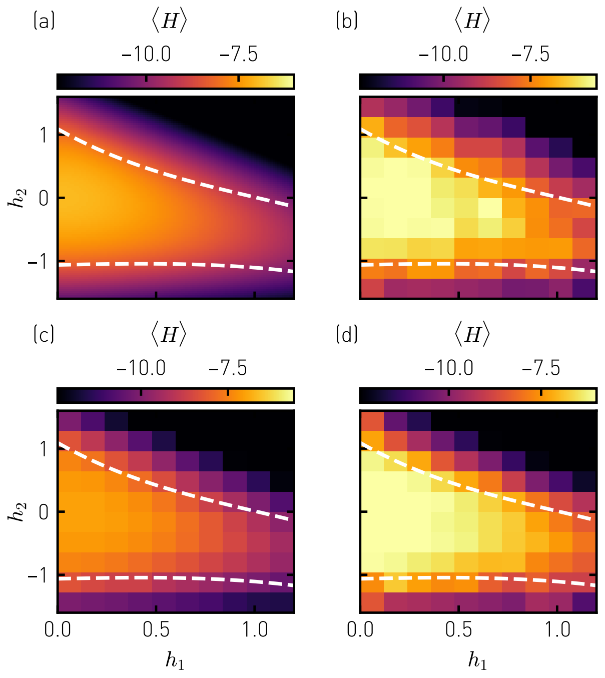

Supplementary Figure 8. **Ground state energy.** Energy expectation value $\langle H \rangle$ with respect to exact ground states in (a), approximate ground states prepared and measured on the quantum hardware in (b), ground states obtained from the ideal state preparation circuit (c) and simulated ground states taking decoherence and gate errors into account in (d).

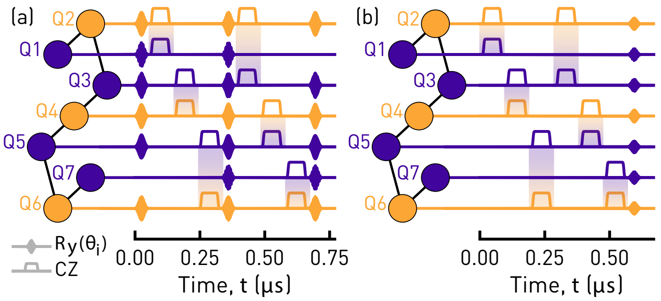

Supplementary Figure 9. Pulse sequences for implementing (a), the variational ground state preparation and (b), the convolutional layer of the QCNN. The qubit colors represent the different qubit idle frequency bands of ~ 5.9 GHz (orange) and ~ 4.2 GHz (purple).

obtained from exact diagonalization of H. The optimization run marked in red color lies above the acceptance threshold of $1 - F > 19\,\%$ (dashed line in Supplementary Fig. 6(b)), which we find to happen more frequently for ground states close to the phase boundary. The challenge of accurately approximating ground states near the phase boundary can be traced back to the diverging correlation length in those parameter regimes, which is also reflected by the larger deviations of $\langle \mathcal{S} \rangle$ from the ideal value (Supplementary Fig. 6(c)) and the larger infidelity (Supplementary Fig. 6(d)). By increasing the variational circuit depth to $m = 2$, the infidelity can be reduced [17], as depicted in Supplementary Fig. 6(d), however under the influence of noise and gate errors, we expect this advantage to diminish and we thus choose to operate our state preparation circuit with $m = 1$.

Previous experiments on superconducting quantum hardware have shown that an exact matrix product state representation of translationally invariant states along a special path in the phase diagram can be used to find exact state preparation circuits [18]. In contrast to this exact method, the variational circuit allows us to approximately prepare any ground state of H with a shallow quantum circuit that is less exposed to noise and gate errors. Under NISQ conditions, finding a balance between these two approaches is crucial to achieve maximal state preparation fidelity.

As discussed in the main text, we reduce the effect of qubit relaxation on the state preparation fidelity by finding equivalent parameters $\tilde{\boldsymbol{\theta}}_{\mathrm{opt}}$, which result in the same unitary $U(\boldsymbol{\theta}_{\mathrm{opt}}) = U(\tilde{\boldsymbol{\theta}}_{\mathrm{opt}})$, but which avoid rotation angles $\tilde{\theta}_i > \pi/2$ in the first layer of single qubit gates [19]. We achieve this by using the identity $R_y(\theta_i + \pi) = R_y(\theta_i)(-iY)$ (see Supplementary Fig. 7(a) and (b)). The additional Y gate can be propagated through the following CZ gates using the identity $CZ(Y \otimes I) = (Y \otimes Z)CZ$ (see Supplementary Fig. 7(c)) and finally be absorbed by the single-qubit rotation in the third layer, yielding the circuit in (d). We repeat this procedure for all angles θ_i in the first layer where $|\theta_i| > \pi/2$. This effectively reduces the probability of being in the excited state and thus the rate of amplitude damping on the qubits. From a Kraus operator simulation of the respective quantum circuits (Supplementary Note 5), we find that using the equivalent angle set $\tilde{\boldsymbol{\theta}}_{\mathrm{opt}}$ can improve the fidelity of the prepared quantum states by up to 20 % compared to using $\boldsymbol{\theta}_{\mathrm{opt}}$.

Ground state energy – As an additional performance measure, complimentary to the fidelity and the string order parameter, we also determine the variational ground state energy in comparison to the exact value, see Supplementary Fig. 8(a). We measure $\langle H \rangle$ by sampling the respective expectation values (see Supplementary Equation (1)) from the state preparation circuit for 10×10 combinations of $\{h_1, h_2\}$. We find the measured values to resemble all qualitative features of the exact energy diagram, compare Supplementary Fig. 8(a) and (b), which additionally confirms the quality of the prepared quantum states also outside the SPT phase where the value of $\langle \mathcal{S} \rangle$ vanishes. In addition, we find good agreement between the exact value of the energy and an ideal simulation of the preparation circuit, compare Supplementary Fig. 8(a) and (c), and most importantly between the measured value of $\langle H \rangle$ and a Kraus operator simulation taking decoherence and two-qubit gate errors into account,

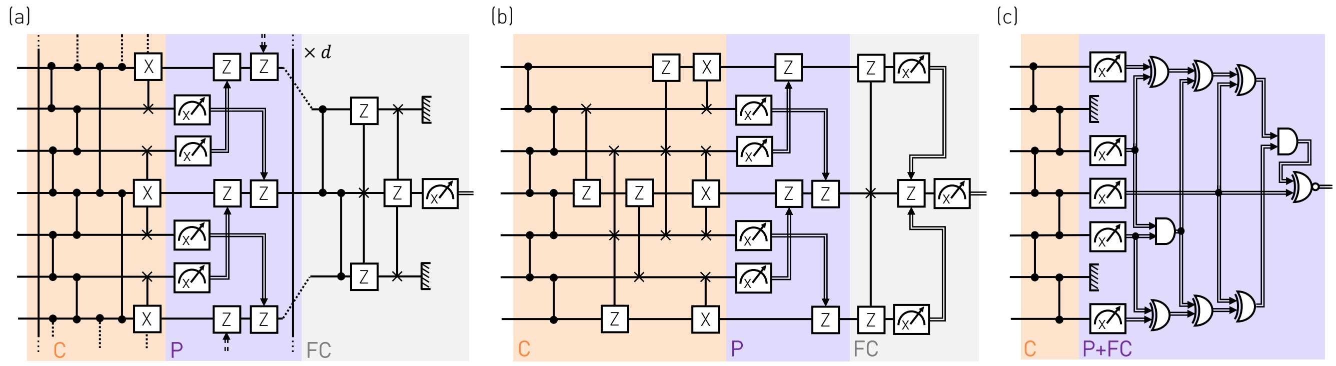

Supplementary Figure 10. **QCNN circuit transformation.** (a) The employed 7-qubit QCNN circuit with quantum gates only (black) and the extension to a larger system size with the QCNN depth d (dashed), which can be transformed into (b) an intermediate 7-qubit circuit and into (c) an equivalent circuit with the pooling and fully connected layer being implemented with classical XOR and AND gates in post-processing.

compare Supplementary Fig. 8(b) and (d). The average difference of the measured energy expectation values $\langle H \rangle$, Supplementary Fig. 8(b), to the energy of the exact ground states, Supplementary Fig. 8(a), evaluates to 1.80.

Kraus operator simulations – To simulate the evolution of a quantum state ρ during the state preparation sequence (Supplementary Fig. 9(a)) and the QCNN sequence (Supplementary Fig. 9(b)) in the presence of decoherence and gate errors, we model each gate as a completely-positive trace preserving (CPTP) process

$$\rho(t+\delta t) = \mathcal{E}(\rho(t)) = \sum_{\alpha=1}^{N} K_\alpha \rho(t) K_\alpha^\dagger, \qquad (4)$$

where δt is the gate duration, K_α are Kraus operators satisfying $\sum_{\alpha=1}^{M} K_\alpha^\dagger K_\alpha = I$ and I is the identity matrix. We compose the circuits as sequences of single-qubit, qubit idling, and two-qubit gate processes.

We describe single-qubit R_y gates by the concatenated process $\mathcal{E}_r(\mathcal{E}_{zz}(\mathcal{E}_y(\rho)))$. Here, $\mathcal{E}_y(\rho) = R_y \rho R_y^\dagger$ is the ideal single-qubit unitary, $\mathcal{E}_{\mathrm{zz}}(\rho) = e^{-i\delta t H_{\mathrm{zz}}}\rho(e^{-i\delta t H_{\mathrm{zz}}})^\dagger$ with $H_{\mathrm{zz}} = \frac{1}{4}\sum_{\langle ij\rangle} \alpha_{zz}^{ij}(I_i - Z_i)(I_j - Z_j)$ describes residual ZZ coupling with strength $\alpha_{\mathrm{zz}}^{ij}$ of qubit i to its neighbors j, and $\mathcal{E}_r(\rho)$ accounts for dephasing and energy relaxation. We model $\mathcal{E}_r$ according to (4) with Kraus operators $K_1 = \sqrt{\gamma_1}\sigma^-$, $K_2 = \sqrt{\gamma_2}Z$ and $K_3 = \sqrt{1-\gamma_2}|0\rangle\langle 0| + \sqrt{1-\gamma_1-\gamma_2}|1\rangle\langle 1|$ with $\gamma_1 = \delta t/T_1$ and $\gamma_2 = \delta t(\frac{1}{2T_2} - \frac{1}{4T_1})$. Here, T_1, and $T_2 = T_2^{\mathrm{e}}$ are taken from experimental characterization experiments, see Tab 1.

We describe the CZ process directly from experimental data by modeling $\mathcal{E}(\rho) = \sum_{\alpha,\beta=1}^{16} \chi_{\alpha\beta} E_\alpha \rho E_\beta^\dagger$ with χ being the process matrix obtained from experimental quantum process tomography (see Supplementary Note 3) and $E_\alpha \in \{I, X, Y, Z\}^{\otimes 2}$. Since CZ gates are executed sequentially, see Supplementary Fig. 9, we use the processes $\mathcal{E}_r$ and $\mathcal{E}_{zz}$ on the remaining idle qubits to take into account decoherence and residual ZZ coupling during the gate time. We implement the Kraus operator simulation of the quantum circuits using the QuTiP Python package [20].

Supplementary Note 5: Properties of the QCNN circuit

QCNN circuit design – The full quantum QCNN circuit as proposed in Ref. [21] involves a convolutional (C) layer, a pooling (P) layer and a fully connected (FC) layer, see Supplementary Fig. 10(a). The C layer performs CZ gates with controls in the computational basis as well as a Toffoli ($\mathrm{C}_x\mathrm{C}_x$NOT) gate and two C_xNOT gates with control in the X basis, which is triggered when control qubits are in the state $|-\rangle$. In the P layer, qubits 2, 3, 5 and 6 are measured in the X basis and Z gates are performed on qubits 1, 4 and 7 when measurements yield $X = -1$.

The design of this particular QCNN is inspired by the MERA representation [22] of the cluster state $|\mathcal{C}\rangle$ and it is constructed such that the cluster state becomes a stable fixed point of the QCNN circuit [21]. The C and P layers transform the cluster state (of 7 qubits) $|\mathcal{C}^{(7)}\rangle \xrightarrow{\mathrm{CP}} |\mathcal{C}^{(3)}\rangle$ to the cluster state $|\mathcal{C}^{(3)}\rangle$ of reduced system size (3 qubits). Perturbations of the input state away from the cluster state can be interpreted as "errors". In analogy to quantum error correction, such errors are detected by measurements in the P layer and corrected by conditional Z gates [21]. In particular, the cluster state perturbed by a single X_i error, for $i = 1, 2, ..., 7$, is mapped onto the unperturbed cluster state, $X_i|\mathcal{C}^{(7)}\rangle \xrightarrow{\mathrm{CP}} |\mathcal{C}^{(3)}\rangle$. This leads to the convergence of states in the SPT phase towards the cluster state, which enables the QCNN to recognize the phase with high fidelity. Such perturbations could either arise in exact ground states due to finite values of h_1 and h_2 or due to errors in the state preparation.

While single Pauli X operations are absorbed by the

C and P layers, single Pauli Z errors propagate through those layers, since $Z_i|\mathcal{C}^{(7)}\rangle \xrightarrow{\text{CP}} Z_j|\mathcal{C}^{(3)}\rangle$. To achieve a high fidelity of detecting the SPT phase in the presence of noise, we extend the originally proposed QCNN circuit from Ref. [21] and design a new FC layer to correct for Z errors. In particular, this new FC layer involves CZ gates with controls in the computational basis, as well as C_xZ gates and a controlled-controlled Z ($\text{C}_x\text{C}_x\text{Z}$) gate with controls in the X basis, which are triggered when control qubits are in the state $|-\rangle$, see Supplementary Fig. 10(a). Finally, qubit 4 is measured in the X basis, which together with the preponed FC layer is equivalent to measuring the observables $\frac{1}{2}(Z_1X_4Z_7 + X_1Z_4 + Z_4X_7 + Y_1X_4Y_7)$ on the state after the C and P layers. For the ideal cluster state $|\mathcal{C}^{(3)}\rangle$ as well as for the cluster state $Z_j|\mathcal{C}^{(3)}\rangle$ perturbed by a single Z_j error on qubit $j = 1, 4$ or 7, this measurement deterministically yields the value 1 and, as a consequence, it effectively corrects the Z error.

For the paramagnetic and the antiferromagnetic state, the QCNN yields the output zero and thus it can be employed for the recognition of the SPT phase. We note that due to finite size effects for the $N = 7$ size of our system, the QCNN output is for all parameter values larger than the SOP $\langle S\rangle$, see Figs. 3b and 3c. This is in contrast to larger system sizes, for which the QCNN output yields a step-like behavior at phase boundaries with vanishing values outside of the SPT phase and near-unity values inside of the SPT phase [21].

Sample Complexity - To investigate the step-like behavior of the QCNN output at phase boundaries in more detail, we perform numerical calculations of the QCNN circuit shown in Supplementary Fig. 10(a) for larger depths up to $d = 3$. For this purpose, we compute ground states of the cluster-Ising Hamiltonian H with $N = 111$ spins using the finite-size density matrix renormalization group (DMRG) [23] with bond dimension $D = 150$. A system of size $N = 111$ spins provides a sufficiently large bulk system for a QCNN circuit of depth $d = 3$ which acts on 55 qubits in the first layer. We additionally verify that increasing the bond dimension to $D = 200$ does not visually change the results presented in Supplementary Fig. 11, confirming a reasonable convergence of our simulation. On the computed ground states we perform the QCNN circuit operations by sequentially applying SWAP and two-qubit unitaries on neighboring sites. We evaluate the QCNN output for depths $d = \{1, 2, 3\}$ from the bulk of the $N = 111$ spin chain and find a step-like behavior at phase boundaries for large circuit depths d, see Supplementary Fig. 11(a). To compare the performance of the QCNN at phase boundaries to the string order parameter $\mathcal{S}$ acting on the same system size $N = 2\cdot 3^d + 1$, we evaluate the sample complexity [21]

$$M = \frac{1.96^2}{\left(\arcsin(\sqrt{p}) - \sqrt{\arcsin(p_0)}\right)^2}, \tag{5}$$

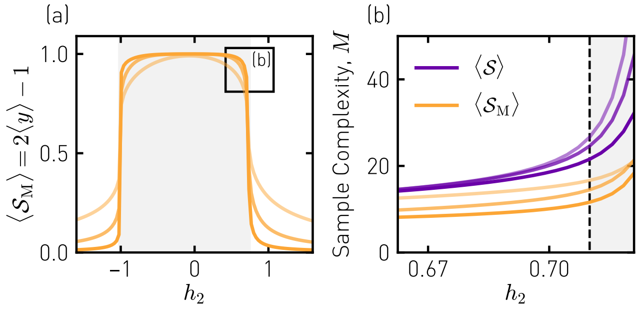

Supplementary Figure 11. **QCNN Sample Complexity.** (a) Numerically calculated output of the QCNN $\langle \mathcal{S}_\text{M}\rangle$ for different circuit depths $d = \{1, 2, 3\}$ (light to dark orange) vs. h_2 and for fixed $h_1 = 0.2$. The shown output values are computed from ground states of system size $N = 111$ found by using finite-size DMRG. The SPT phase (gray) is determined from sharp peaks in the second derivative of the ground state energy $\partial^2\langle H\rangle/\partial h_2^2$ found by infinite-size DMRG. (b) Sample complexity computed from the values shown in (a) compared to the sample complexity of the SOP $\langle \mathcal{S}\rangle$. The numerically calculated results in the gray region can be affected significantly by finite-size effects, since the correlation length exceeds the system size in this parameter regime.

with $p_0 = 0.5$ and $p = (\langle \mathcal{S}\rangle + 1)/2$ for the string order parameter and $p = (\langle \mathcal{S}_\text{M}\rangle + 1)/2$ for the QCNN output. Here we find a similar improvement in sample complexity for the proposed QCNN circuit as was found in Ref. [21], see Supplementary Fig. 11(b). In our experiment, we implement part of the QCNN circuit classically, which in our particular example is possible for any circuit depth d, and therefore the improvement in sample complexity M is also accessible with the circuit modifications presented in the following section.

Equivalent QCNN circuit – Here we derive a circuit that is equivalent to the full quantum QCNN circuit shown in Supplementary Fig. 10(a) but features fewer quantum gates, a projective measurement and classical post-processing, see Supplementary Fig. 10(c). We use a notation, where CZ_{jk} denotes a CZ gate acting symmetrically on qubits j and k, and $\text{C}_x\text{NOT}_{j;k}$ denotes a controlled not gate acting on qubit k controlled by qubit j in the X basis ($\text{C}_x\text{C}_x\text{NOT}_{jl;k}$ denotes a controlled-controlled not gate acting on qubit k controlled by qubits j and l in the X basis).

We first note that CZ_{14} and CZ_{47} gates are performed twice in the full QCNN circuit, see Supplementary Fig. 10(a), namely the first pair in the C layer and the second pair in the FC layer. The second pair commutes with the P layer and thus it can be moved from the FC layer to the C layer. As the CZ gates do not commute with the Toffoli gate $\text{C}_x\text{C}_x\text{NOT}_{35;4}$, we use the identity $\text{CZ}_{47}\text{C}_x\text{C}_x\text{NOT}_{35;4} = \text{C}_x\text{C}_x\text{NOT}_{35;4}\text{C}_x\text{C}_x\text{Z}_{35;7}\text{CZ}_{47}$ and an analogous identity for CZ_{14} gate, to exchange the second pair of CZ gates and the Toffoli gate by introducing

C_xC_xZ gates with controls on qubits 3 and 5 in the X basis. We use this identity in an analogue fashion to exchange $C_x\mathrm{NOT}_{2;1}$ and $C_x\mathrm{NOT}_{6;7}$. Since $\mathrm{CZ}^2 = I$, the second pair of CZ gates moved from the FC layer cancels with the first pair in the C layer and we obtain the equivalent circuit shown in Supplementary Fig. 10(b). Note that we also replaced the last gate of the FC layer, the C_xC_xZ gate, by equivalent measurements of qubits 1 and 7 in the X basis and the Z_4 gate conditioned on measuring $X_1 = X_7 = -1$.

We will now show that in the equivalent quantum circuit depicted in Supplementary Fig. 10(c), all gates except the first two layers of CZ gates, with controls in the computational basis, can be performed in classical post-processing following the projective measurement of all qubits in the X basis. The Toffoli gate is directly followed by the measurement of the control qubits 3 and 5 and it anticommutes with the remaining Z_4 gates before the measurement of qubit 4. As a result, the Toffoli gate can produce only an undetectable global phase factor -1 and thus it can be removed. Z gates produce a phase flip $|\pm\rangle \to |\mp\rangle$, which can be implemented in classical post-processing after the measurement in the X basis with eigenstates $|\pm\rangle$. Conditional Z gates correspond to XOR gates in post-processing. Controlled Z gates with controls in the X basis, which are directly followed by the measurement of control qubits, are equivalent to conditional Z gates and thus they can also be analogously implemented in post-processing. Moving all conditional Z gates and controlled Z gates with controls in the X basis, one by one, into post-processing, we obtain the equivalent circuit shown in Supplementary Fig. 10(c). This circuit is equivalent to the full quantum QCNN circuit but involves only two layers of CZ gates, projective measurement and classical post-processing.

Note that a full quantum QCNN circuit of any depth d can be reduced to two layers of CZ gates and classical post-processing. This can be shown using the same gate identities as for the depth $d = 1$ circuit. Specifically, the third and the fourth layers of CZ gates of the fth layer – performed on qubits that are not measured in the subsequent P layer, and present in the C layer – is the inverse unitary to the first and the second layers of CZ gates from the $(f + 1)$th layer, see Supplementary Fig. 10(a). Moving the CZ gates from the $(f + 1)$th layer to the fth layer cancels these 4 layers of CZ gates, leaving only the P layer plus some additional C_xC_xZ gates. By canceling these CZ gates in every layer we obtain a circuit that involves only two layers of CZ gates at the beginning followed by C_xC_xX, C_xC_xZ, and C_xZ gates. All of these gates are Pauli gates with controls in X-bases and thus can be implemented in classical post-processing for any depth d circuit.

Multi-scale string order parameter – The observable measured at the output of the FC layer corresponds to measuring a multi-scale string order parameter of the form

$$\mathcal{S}_{\mathrm{M}} = \sum_{jk} \eta^{(1)}_{jk} \mathcal{S}_{jk} + \sum_{jklm} \eta^{(2)}_{jklm} \mathcal{S}_{jk}\mathcal{S}_{lm} + ..., \tag{6}$$

where

$$\mathcal{S}_{jk} = Z_j X_{j+1} X_{j+3} ... X_{k-3} X_{k-1} Z_k, \tag{7}$$

are string order parameters of varying length and $\eta^{(\alpha)}$ are coefficients weighting the individual terms [21]. We now determine the multi-scale SOP measured by the QCNN implemented in the experiment. We choose the termination at the edges of the circuit as shown in Supplementary Fig. 10(a). For d layers of convolution and pooling this choice corresponds to $N = 2 \cdot 3^d + 1$ qubits at the input of the QCNN. For the analysis of the full QCNN circuit, depicted in Supplementary Fig. 10(a), we replace measurements and conditional unitaries in the P layers by controlled Z gates with controls in the X basis. In this way, we can represent the QCNN circuit by a unitary $U = U_{\mathrm{FC}}V$, where $V = U^{(d)}_{\mathrm{CP}}...U^{(1)}_{\mathrm{CP}}$ and $U^{(f)}_{\mathrm{CP}} = U^{(f)}_{\mathrm{P}} U^{(f)}_{\mathrm{C}}$, consisting of d convolutional $U^{(f)}_{\mathrm{C}}$ and pooling $U^{(f)}_{\mathrm{P}}$ layers, $f = 1, 2, \ldots, d$, as well as the fully connected layer U_{FC}, which represent the unitary operations of the respective circuit layers shown in Supplementary Fig. 10(a).

The outcome of measuring the output qubit with index $i = \frac{N+1}{2}$ in the FC layer of the QCNN corresponds to the expectation value of the multi-scale SOP

$$\mathcal{S}_{\mathrm{M}} = V^\dagger U^\dagger_{\mathrm{FC}} X_{\frac{N+1}{2}} U_{\mathrm{FC}} V = \frac{1}{2}\left(\mathcal{S}^{I}_{\mathrm{M}} - \mathcal{S}^{II}_{\mathrm{M}}\right), \tag{8}$$

where

$$\mathcal{S}^{I}_{\mathrm{M}} = V^\dagger \left(C^{(d)}_{\frac{N+1}{2}-3^d} + C^{(d)}_{\frac{N+1}{2}} + C^{(d)}_{\frac{N+1}{2}+3^d} \right) V, \tag{9}$$

$$\mathcal{S}^{II}_{\mathrm{M}} = V^\dagger C^{(d)}_{\frac{N+1}{2}-3^d} C^{(d)}_{\frac{N+1}{2}} C^{(d)}_{\frac{N+1}{2}+3^d} V, \tag{10}$$

with $C^{(f)}_j = Z_{j-3^f} X_j Z_{j+3^f}$. To explicitly evaluate the multi-scale SOP, we use the recursive relation

$$\begin{aligned} U^{(f)\,\dagger}_{\mathrm{CP}} &\left(C^{(f)}_j C^{(f)}_{j+2\cdot 3^f} ... C^{(f)}_k \right) U^{(f)}_{\mathrm{CP}} \\ &= L^{(f-1)}_j \left(C^{(f-1)}_j C^{(f-1)}_{j+2\cdot 3^{f-1}} ... C^{(f-1)}_k \right) R^{(f-1)}_k, \end{aligned} \tag{11}$$

where $k \geq j$ and

$$L^{(f)}_j = \frac{1}{2}\left(C^{(f)}_{j-4\cdot 3^f} C^{(f)}_{j-2\cdot 3^f} - C^{(f)}_{j-4\cdot 3^f} + C^{(f)}_{j-2\cdot 3^f} + 1 \right), \tag{12}$$

$$R^{(f)}_j = \frac{1}{2}\left(1 + C^{(f)}_{j+2\cdot 3^f} - C^{(f)}_{j+4\cdot 3^f} + C^{(f)}_{j+2\cdot 3^f} C^{(f)}_{j+4\cdot 3^f} \right). \tag{13}$$

Terms at zero depth, i.e. measured at the input state, and at the edge of the array reduce to $L^{(0)}_1 = L^{(0)}_4 = R^{(0)}_{N-3} = R^{(0)}_N = 1$ and we use the notation $X_j = Z_j = \mathbb{1}$

$\eta^{(\alpha)}$	Product	Pauli string						
$+1/4$	$\mathcal{S}_{02}$	X_1	Z_2					
$+1/4$	$\mathcal{S}_{04}$	X_1		X_3	Z_4			
$+1/4$	$\mathcal{S}_{06}$	X_1		X_3		X_5	Z_6	
$+1/4$	$\mathcal{S}_{28}$		Z_2	X_3		X_5		X_7
$+1/4$	$\mathcal{S}_{48}$				Z_4	X_5		X_7
$+1/4$	$\mathcal{S}_{68}$						Z_6	X_7
$-1/4$	$\mathcal{S}_{02}\mathcal{S}_{46}$	X_1	Z_2		Z_4	X_5	Z_6	
$-1/4$	$\mathcal{S}_{24}\mathcal{S}_{68}$		Z_2	X_3	Z_4		Z_6	X_7
$+1/2$	$\mathcal{S}_{35}$			Z_3	X_4	Z_5		
$-1/2$	$\mathcal{S}_{08}\mathcal{S}_{35}$	X_1		Y_3	X_4	Y_5		X_7

Supplementary Table 3. Individual products of SOPs involved in the multi-scale string order parameter $\mathcal{S}_M$ (18) measured by the employed 7-qubit QCNN circuit from Supplementary Fig. 10(a) with $d = 1$. The terms are sorted and subdivided according to their measurement basis.

for $j > N$ and $j < 1$. Using the recursive relation (11) d times, we obtain the explicit form (6) of the multi-scale SOP $\mathcal{S}_M$ from Supplementary Equation (8). To evaluate the second part $\mathcal{S}_{\mathrm{M}}^{II}$ of the multi-scale SOP, we exploit that individual $C_i^{(d)}$ terms commute to express $\mathcal{S}_{\mathrm{M}}^{II} = V^\dagger C^{(d)}_{\frac{N+1}{2}} V \cdot V^\dagger C^{(d)}_{\frac{N+1}{2}-3^d} C^{(d)}_{\frac{N+1}{2}+3^d} V$ and use the recursive relation (11) separately for $C^{(d)}_{\frac{N+1}{2}}$ and $C^{(d)}_{\frac{N+1}{2}-3^d} C^{(d)}_{\frac{N+1}{2}+3^d}$.

We can estimate the scaling in terms of the number of SOP products in (6) by considering products of non-overlaping SOPs (NSOPs). The general form of a single NSOP is given in (7), however when considering the number of products there is an ambiguity since

$$\mathcal{S}_{jk} = Z_j X_{j+1} \cdots X_{k-1} Z_k = \prod_{n=1}^{\frac{k-j}{2}-1} \mathcal{S}_{j+2n-2,j+2n}. \quad (14)$$

Therefore we define a product of n NSOPs as the product of n different NSOPs where there is no two NSOPs that overlap. Furthermore, for a product of n NSOPs we require that there is at least a gap of 1 qubit between the support of neighboring NSOPs, we label a term that satisfies both these conditions by $(\mathbf{n})$. These conditions are both preserved by the recursion relation (11). The second condition for a product of NSOPs is thus motivated by the recursion relation and means that a product of NSOPs will give rise to a sum of products of NSOPs under this recursion. Relation (11), thus maps a single NSOP to 9 different single NSOPs, 6 different products of two NSOPs, and a single product of three NSOPs. We can denote this by the map $(\mathbf{1}) \mapsto 9(\mathbf{1}) + 6(\mathbf{2}) + 1(\mathbf{3})$. Since the recursion relation (11) preserves the spacing our recursion relation can be expressed as

$$\begin{aligned}(\mathbf{n}) &\mapsto [9(\mathbf{1}) + 6(\mathbf{2}) + 1(\mathbf{3})]^{\otimes n} \\ &= \sum_{a=0}^{n} \sum_{b=0}^{n-a} \frac{n!}{a!b!(n-a-b)!} 3^{2n-2a-b} 2^b \,(\mathbf{n}{+}\mathbf{2a}{+}\mathbf{b})\end{aligned} \quad (15)$$

Analyzing this we know that after f repetitions that started with a product of n NSOPs we will get a single term that is a product of $3^f n$ NSOPs, labeled $(\mathbf{3^f n})$. Then if we apply the recursion relation one further time we will obtain at least $16^{3^f n}$ separate terms, placing a lower bound on the number of SOP products that must be measured after $f+1$ layers. Furthermore, for $f = d-1$ or $f = d-2$ this indicates that the number of SOP products will scale double-exponentially in the QCNN depth d.

As a complete example for the $d = 1$ case, the output of the QCNN implemented in the experiment with $N = 7$ qubits corresponds to the expectation value of the multi-scale SOP

$$\begin{aligned}\mathcal{S}_{\mathrm{M}} &= U^\dagger_{\mathrm{CP}} U^\dagger_{\mathrm{FC}} X_4 U_{\mathrm{FC}} U_{\mathrm{CP}} && (16)\\ &= \frac{1}{2} U^\dagger_{\mathrm{CP}} (C_1^{(1)} + C_4^{(1)} + C_7^{(1)} - C_1^{(1)} C_4^{(1)} C_7^{(1)}) U_{\mathrm{CP}} && (17)\\ &= \sum_{jk} \eta^{(1)}_{jk} \mathcal{S}_{jk} + \sum_{jklm} \eta^{(2)}_{jklm} \mathcal{S}_{jk} \mathcal{S}_{lm} && (18)\end{aligned}$$

By evaluating the unitary transformation U_{CP}, we obtain the explicit form (18) of the multi-scale SOP with products of SOPs $\mathcal{S}_{jk}$ weighted with coefficients $\eta^{(\alpha)}$ which are listed in Supplementary Table 3. Due to the small system size and the shallow depth $d = 1$, the total number of terms measured by the QCNN reduces to 10.

Direct measurement of QCNN output – Instead of performing the QCNN, we could determine the expectation value of the multi-scale SOP $\mathcal{S}_{\mathrm{M}}$ (6) by directly measuring the individual products of SOPs on the input state to obtain the same outcome. We now discuss a crucial reduction in the number of measurements provided by the QCNN compared to the direct sampling from the input state. This in turn leads to a reduction in the number of input-state copies required for determining the expectation value of the multi-scale SOP $\mathcal{S}_{\mathrm{M}}$ since for each projective measurement we need to prepare a separate copy of the input state. In the direct measurement, sampling from multiple measurement bases is required, each basis requiring that we prepare an independent copy of the input state.

We have already shown that the number of products of SOPs in the multi-scale SOP $\mathcal{S}_{\mathrm{M}}$ scales double-exponentially with the QCNN circuit depth d. A single projective measurement of the input state consists of measuring all qubits in a local basis (X, Y or Z basis). We can use classical post-processing to determine the expectation value of several products of SOPs from the same measurement. In particular, two products of SOPs can be sampled from the same measurement if they involve the same Pauli operator on all qubits, on which both products of SOPs act non-trivially. A product of SOPs acts trivially (non-trivially) on qubit i if it involves the identity I_i (the Pauli X_i, Y_i or Z_i operator).

The QCNN proposed in Ref. [21] measures only the first part, $\mathcal{S}_{\mathrm{M}}^I$, of the multi-scale SOP $\mathcal{S}_{\mathrm{M}}$, see Supplemen-

tary Equation (9). The recursive relation (11) dictates that, for an arbitrary depth d of the QCNN, all products of SOPs involved in $\mathcal{S}_{\mathrm{M}}^{I}$ exhibit either (i) the Pauli X operator at even qubits and Pauli Z operator or identity at odd qubits, or (ii) the Pauli X operator at odd qubits and Pauli Z operator or identity at even qubits. As a result, using classical post-processing, we can determine the expectation values of all products of SOPs involved in $\mathcal{S}_{\mathrm{M}}^{I}$ from only two different measurements. Namely (i) the measurement of all even qubits in the X basis and all odd qubits in the Z basis, and (ii) the measurement of all odd qubits in the X basis and all even qubits in the Z basis. This shows that the output of the QCNN proposed in Ref. [21] can be efficiently sampled directly from the input state. The direct sampling requires only twice as many measurements as sampling after performing that QCNN with an arbitrary depth.

The lack of an efficient sampling method when using direct measurements on the input state is therefore due to the modified FC layer that we considered in our work. The second term, $\mathcal{S}_{\mathrm{M}}^{II}$, splits into $V^\dagger C^{(d)}_{\frac{N+1}{2}} V$ and $V^\dagger C^{(d)}_{\frac{N+1}{2}-3^d} C^{(d)}_{\frac{N+1}{2}+3^d} V$. If we let the $V^\dagger C^{(d)}_{\frac{N+1}{2}} V$ term produce the 16^{3^f} NSOPs as described in the last subsection, this could by itself be efficiently sampled. Here, however $V^\dagger C^{(d)}_{\frac{N+1}{2}-3^d} C^{(d)}_{\frac{N+1}{2}+3^d} V$ will at the same time generate a product of Pauli-X terms on all odd qubits. Hence, for all the 16^{3^f} products, the odd sites must now be measured in either the X- or Y-basis rather than being ignored or measured in the Z-basis. A cursory analysis of the 16 terms that appear in (11) shows that no pair would agree on all qubits regarding which would have Pauli-Zs present. This means that, when we include the chain of Pauli-Xs from $V^\dagger C^{(d)}_{\frac{N+1}{2}-3^d} C^{(d)}_{\frac{N+1}{2}+3^d} V$, no pair of the 16 terms can be simutanously measured. Since each of the 3^f NSOPs in the product we are using expands independently, this gives us 16^{3^f} products of SOPs that will pairwise disagree on which basis should be measured in for at least one qubit.

A lower bound to the number of terms that need to be measured individually in direct sampling from the input state can then be found by starting from the smallest single chain term, $C^{(d-1)}_{\frac{N+1}{2}} = Z_{\frac{N+1}{2}-3^{d-1}} X_{\frac{N+1}{2}} Z_{\frac{N+1}{2}+3^{d-1}}$, generated from $U^{(d)\dagger}_{CP} C^{(d)}_{\frac{N+1}{2}} U^{(d)}_{CP}$ and working back to the input layer. Considering this lower bound avoids the need for extra discussions of boundary effects that would emerge when counting all appearing terms. The lower bound is then obtained by taking $f = d - 2$ in 16^{3^f} and shows that there are at least $16^{3^{d-2}}$ products of NSOPs that are pairwise not simultaneously measurable.

As a result, the number of direct measurements on the input state increases double-exponentially with the depth of the QCNN, or (at the largest depth) exponentially in the number of qubits. This renders the direct sampling of the QCNN output unfeasible. On the other hand, performing the QCNN enables us to efficiently sample the multi-scale SOP $\mathcal{S}_{\mathrm{M}}$ from a single measurement of the single qubit at the output of the QCNN (or equivalently, by the equivalent constant depth circuit and post-processing).

[1] C. K. Andersen, A. Remm, S. Lazar, S. Krinner, N. Lacroix, G. J. Norris, M. Gabureac, C. Eichler, and A. Wallraff, Nature Physics **16**, 875 (2020).

[2] C. C. Bultink, B. Tarasinski, N. Haandbæk, S. Poletto, N. Haider, D. J. Michalak, A. Bruno, and L. DiCarlo, Appl. Phys. Lett. **112**, 092601 (2018).

[3] J. Heinsoo, C. K. Andersen, A. Remm, S. Krinner, T. Walter, Y. Salathé, S. Gasparinetti, J.-C. Besse, A. Potočnik, A. Wallraff, and C. Eichler, Phys. Rev. Appl. **10**, 034040 (2018).

[4] C. Macklin, K. O'Brien, D. Hover, M. E. Schwartz, V. Bolkhovsky, X. Zhang, W. D. Oliver, and I. Siddiqi, Science **350**, 307 (2015).

[5] F. Motzoi, J. M. Gambetta, P. Rebentrost, and F. K. Wilhelm, Phys. Rev. Lett. **103**, 110501 (2009).

[6] M. C. Collodo, J. Herrmann, N. Lacroix, C. K. Andersen, A. Remm, S. Lazar, J.-C. Besse, T. Walter, A. Wallraff, and C. Eichler, Phys. Rev. Lett. **125**, 240502 (2020).

[7] S. Krinner, P. Kurpiers, B. Royer, P. Magnard, I. Tsitsilin, J.-C. Besse, A. Remm, A. Blais, and A. Wallraff, Phys. Rev. Appl. **14**, 044039 (2020).

[8] L. DiCarlo, J. M. Chow, J. M. Gambetta, L. S. Bishop, B. R. Johnson, D. I. Schuster, J. Majer, A. Blais, L. Frunzio, S. M. Girvin, and R. J. Schoelkopf, Nature **460**, 240 (2009).

[9] F. W. Strauch, P. R. Johnson, A. J. Dragt, C. J. Lobb, J. R. Anderson, and F. C. Wellstood, Phys. Rev. Lett. **91**, 167005 (2003).

[10] J. Lisenfeld, A. Bilmes, A. Megrant, R. Barends, J. Kelly, P. Klimov, G. Weiss, J. M. Martinis, and A. V. Ustinov, npj Quantum Information **5**, 105 (2019).

[11] P. V. Klimov, J. Kelly, Z. Chen, M. Neeley, A. Megrant, B. Burkett, R. Barends, K. Arya, B. Chiaro, Y. Chen, A. Dunsworth, A. Fowler, B. Foxen, C. Gidney, M. Giustina, R. Graff, T. Huang, E. Jeffrey, E. Lucero, J. Y. Mutus, O. Naaman, C. Neill, C. Quintana, P. Roushan, D. Sank, A. Vainsencher, J. Wenner, T. C. White, S. Boixo, R. Babbush, V. N. Smelyanskiy, H. Neven, and J. M. Martinis, Phys. Rev. Lett. **121**, 090502 (2018).

[12] M. A. Rol, F. Battistel, F. K. Malinowski, C. C. Bultink, B. M. Tarasinski, R. Vollmer, N. Haider, N. Muthusubramanian, A. Bruno, B. M. Terhal, and L. DiCarlo, Phys. Rev. Lett. **123**, 120502 (2019).

[13] V. Negirneac, H. Ali, N. Muthusubramanian, F. Battistel, R. Sagastizabal, M. S. Moreira, J. F. Marques, W. J. Vlothuizen, M. Beekman, C. Zachariadis, N. Haider, A. Bruno, and L. DiCarlo, Phys. Rev. Lett. **126**, 220502 (2021).

[14] M. A. Rol, L. Ciorciaro, F. K. Malinowski, B. M. Tarasin-

ski, R. E. Sagastizabal, C. C. Bultink, Y. Salathe, N. Haandbaek, J. Sedivy, and L. DiCarlo, Appl. Phys. Lett. **116**, 054001 (2020).

[15] I. L. Chuang and M. A. Nielsen, J. Mod. Opt. **44**, 2455 (1997).

[16] H. Abraham and et al., "Qiskit: An Open-source Framework for Quantum Computing," (2021).

[17] C. Bravo-Prieto, J. Lumbreras-Zarapico, L. Tagliacozzo, and J. I. Latorre, Quantum **4**, 272 (2020).

[18] A. Smith, B. Jobst, A. G. Green, and F. Pollmann, Phys. Rev. Research **4**, L022020 (2022).

[19] E. Fontana, M. Cerezo, A. Arrasmith, I. Rungger, and P. J. Coles, arXiv:2011.08763 (2020).

[20] J. R. Johansson, P. D. Nation, and F. Nori, Comput. Phys. Commun. **184**, 1234 (2013).

[21] I. Cong, S. Choi, and M. D. Lukin, Nature Physics **15**, 1273 (2019).

[22] G. Vidal, Phys. Rev. Lett. **101**, 1110501 (2008).

[23] J. Hauschild and F. Pollmann, SciPost Phys. Lect. Notes (2018).
